# Bioinspired Strategies for Directional Water Transport in Asymmetric Membranes

**DOI:** 10.1021/acsapm.6c00561

**Published:** 2026-04-27

**Authors:** Luca Grillo, Natthakan Rattanaphong, Pornsuda Kotcharat, Bhumin Than-Ardna, Hathaikarn Manuspiya, Stephan Thierry Dubas, Christoph Weder

**Affiliations:** † Adolphe Merkle Institute, Polymer Chemistry and Materials, 27211 University of Fribourg, Chemin des Verdiers 4, Fribourg 1700, Switzerland; ‡ The Petroleum and Petrochemical College, 26683 Chulalongkorn University, Bangkok 10330, Thailand; § Center of Excellence on Petrochemical and Materials Technology, Bangkok 10330, Thailand; ∥ Machine Learning for Polymers and Materials Discovery Research Unit, The Petroleum and Petrochemical College, Chulalongkorn University, Bangkok 10330, Thailand

**Keywords:** dense membranes, directional permeation, water transport, bioinspiration, water-induced plasticization, asymmetric membranes

## Abstract

The directional transfer of water is an important process for many living organisms and an increasingly important technological concept that enables applications such as fog harvesting, separation processes, smart packaging, and advanced textiles. Among the various strategies for achieving directional permeation, asymmetric dense polymer membranes have proven particularly effective. Over the past decade, research on natural and engineered systems has expanded significantly, leading to numerous innovative material solutions. After providing a historical perspective on research on directional biological membranes, this review summarizes the fundamentals governing asymmetric permeation in dense polymer membranes, highlights recent advances concerning the design, fabrication, and characterization of such structures, and discusses possible applications. Finally, current challenges and future opportunities for advancing this field toward scalable and multifunctional systems are presented.

## Graphical abstract

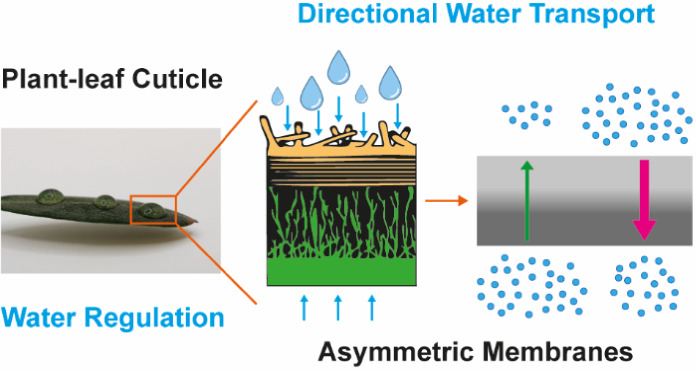

## 1 Directional Water Transport in Biological Systems

The directional transport of water is one of many phenomena observed in nature that have inspired the design of artificial materials with similar functionality.^1–4^ Indeed, many biological organisms exploit mechanisms of directional transport to manage water supply and retention.^5^ For example, after the evolutionary process of adaptation to warm, dry environments, various desert plant and animal species are able to harvest water from fog events.^4,6–8^ Fog-harvesting solutions in natural organisms often rely on hierarchical structures or specialized wettable surfaces that guide water flow toward absorption or storage sites,^7–9^ minimizing evaporation losses.^6^

Many examples of directional water transport rely on specific wetting phenomena that promote the movement of *liquid* water along a preferred direction.^10^ Over the past two decades, several biological systems^6–14^ with directional wetting abilities have garnered significant attention as blueprints for bioinspired materials and devices for fog harvesting^15–18^ and other applications.^5,19–21^ A comprehensive review of biological organisms capable of transporting *liquid* water in a directional manner has recently been provided by Gurera and Bhushan,^6^ while recent advancements in biomimetic systems inspired by this natural feature have been discussed by Yu et al.^4^ as well as Ma and Dong.^1^

In addition to the directional wetting and surface-mediated liquid transport, some biological membranes exhibit directional water transport properties in terms of asymmetric permeation, i.e., diffusional transport, or “passive transport” as referred to by Schultz.^22^ This distinction is particularly relevant in the context of membrane-based systems, where directionality arises not from guided liquid motion along a surface, but from anisotropic transport across a barrier. In this context, directional water permeation describes a condition in which the magnitude of the permeation flow varies depending on which side of the membrane is exposed to the higher water activity. Of course, the direction of water vapor transport always follows the water vapor pressure (or water activity) gradient. An example of a biological membrane exhibiting such anisotropic transport behavior is the cuticle, a protective layer that many plants and insects have developed to, among other functions, regulate mass transport with the environment.^23–28^

Building on these biological examples, this review first examines directional transport through cuticles, focusing on plant cuticles as model natural membranes exhibiting asymmetric water permeation. It then addresses the theoretical framework governing directional water transport through asymmetric polymer membranes and surveys recent progress in the development of artificial dense (i.e., nonporous) membranes with directional water-transport properties, highlighting their potential for technologically relevant applications. Finally, current challenges and future opportunities for advancing the field are discussed, with particular emphasis on the importance of a closer integration of theoretical understanding and experimental realization to fully realize the potential of directional water transport in dense membranes.

Directional water transport across cuticular membranes was first reported in 1941 by Hurst, who found that water evaporation through cuticles isolated from *Calliphora* larvae changes depending on which side of the membrane is exposed to water.^29^ The evaporation rate in the inward direction was reported to exceed that of the opposite direction by a factor of 100, a magnitude that is difficult to reconcile with the current understanding of the process (*vide infra*). Hurst attributed this anisotropy to the compositionally asymmetric structure of the insect cuticle, which features an inner hydrophilic layer that consists of a mixture of protein and chitin and a hydrophobic lipid outer layer, the epicuticle.^29^ Hurst later hypothesized that a valve mechanism is at play in the epicuticle, whose water permeability changes in response to variations in the external environment’s humidity.^30^ The proposed valve system was thought to open when the outer epicuticular layer is hydrated, allowing water uptake, while exposure to dry air and dehydration would close the valve, thus limiting water loss.^30^ In the discussion of these findings, Beament reported that the cuticle of another insect species he examined, i.e., *Rhodnius prolixus*, also exhibited asymmetric transport properties, with water also being transferred more efficiently in the physiological inward direction.^31^ Hartley provided a theoretical explanation of this phenomenon, attributing it to the mathematical condition that the diffusion coefficient varies within a membrane, depending on both, spatial position and permeant concentration.^31^ This theoretical framework was later rigorously elaborated and experimentally validated in artificial multilayer polymeric membranes by Rogers and colleagues,^32^ as discussed below.

Building upon the early transport studies of Hurst and Beament,^29–31^ Richards and coworkers undertook a detailed examination of the “asymmetrical penetration” of both water and heavy water through the cuticular membranes isolated from *Sarcophaga bullata* larvae.^33^ Their findings confirmed that the penetration rate was consistently higher when water entered from the epicuticle, with asymmetry values in good agreement with those reported by Beament.^31,33^ While the previous experiments were all carried out with the cuticles placed between a wet donor and a dry receiver compartment, Richards et al. also investigated the transport properties of the insect cuticles when both sides were hydrated. This was accomplished using heavy water as a tracer. Interestingly, under these conditions, no asymmetry was observed, i.e., the penetration rates in the “inward” and “outward” directions were equal.^33^ Based on these findings, the authors rejected Hurst’s hypothesis concerning the existence of a valve mechanism at play in the epicuticle and concluded that most likely the observed asymmetrical penetration is the result of a complex mathematical condition arising from the superimposition of layers of different composition within the cuticle, wherein the permeability is a function of both the dry composition and the extent of swelling, thereby corroborating the hypothesis proposed by Hartley.^30,31,33^ Although asymmetric water transport across the cuticle has been documented for multiple insect species,^29–31^ Richards and coworkers expressed their doubts about its ecological relevance.^33^ However, recent studies have documented that insect cuticle hydration is important for preserving mechanical properties and suggest that it may also constitute an important mechanism for water regulation.^25,34,35^

Regulation of water transport has been more widely reported for plant cuticles, which constitute the primary protective barrier of most aerial parts of land plants.^28,36–39^ In combination with stomata, which are microscopic pores typically located on the lower surface of leaves (i.e., the abaxial side) or on other aerial organs, such as stems and flowers, the cuticle regulates water exchange and the transportation of gaseous and liquid substances between the plant and its surroundings, with water transport proposed to involve contributions from both lipophilic and polar pathways.^26–28,37,39–43^

From a materials science perspective, plant cuticles are dense composite membranes featuring a compositionally graded structure, as illustrated in Figure 1.^26,28,37–40,44^ Although cuticles from different plant species vary in structure and composition,^44,45^ their organization is similar.^44^ The primary component is an amorphous matrix of cutin, a cross-linked polyester in which hydroxy- and/or epoxy-functionalized C_16_ and C_18_ fatty acids constitute the primary building blocks.^26,28,38–40,46^ On the inner cuticular side, cutin is typically mixed with a fibrous polysaccharide network, mainly composed of pectin and cellulose, originating from the wall of epidermal cells.^26,39,40^ On the outer surface, the cuticle also contains soluble waxes (mainly C_20_–C_40_ derivatives of *n*-acyl alkanes)^26,39^ either within the cutin layer (intracuticular waxes) or on the surface (epicuticular waxes), where they usually assemble into complex three-dimensional crystalline structures.^26,38–40^ These hydrophobic waxes serve as the primary water barrier, and their removal has been reported to increase the water permeability by 2–4 orders of magnitude.^40,47^ Beyond their multicomponent composition, plant cuticles exhibit pronounced heterogeneity in the transverse direction, a graded structure that has been correlated with the multiple functional roles these biological membranes play in sustaining plant life.^39^

**Figure 1:**
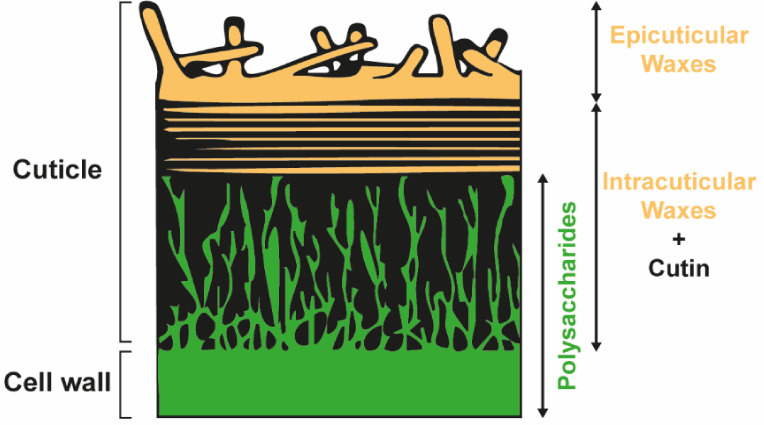
Schematic representation of the cross-section of a plant cuticle. The schematic was redrawn based on ref 44.

The main functions of plant cuticles are summarized in Figure 2. In addition to regulating the transport of water and other gases and solutes (Figure 2a),^27,40,47^ cuticles also affect surface properties such as wettability,^48–50^ antiadhesiveness,^51–53^ and light-screening (Figure 2b–d).^54–56^ Serving as the primary interface with the external environment, cuticles mediate interactions with biotic stresses, such as insects and microorganisms (Figure 2e),^36,57,58^ while ensuring the mechanical resistance of plant tissue (Figure 2f).^59–61^ As this review is focused on directional transport, interested readers are referred to the works of Yeats and Rose,^28^ Bargel et al.,^39^ and Koch and coworkers for a comprehensive discussion of the role of plant cuticles.^62^

**Figure 2:**
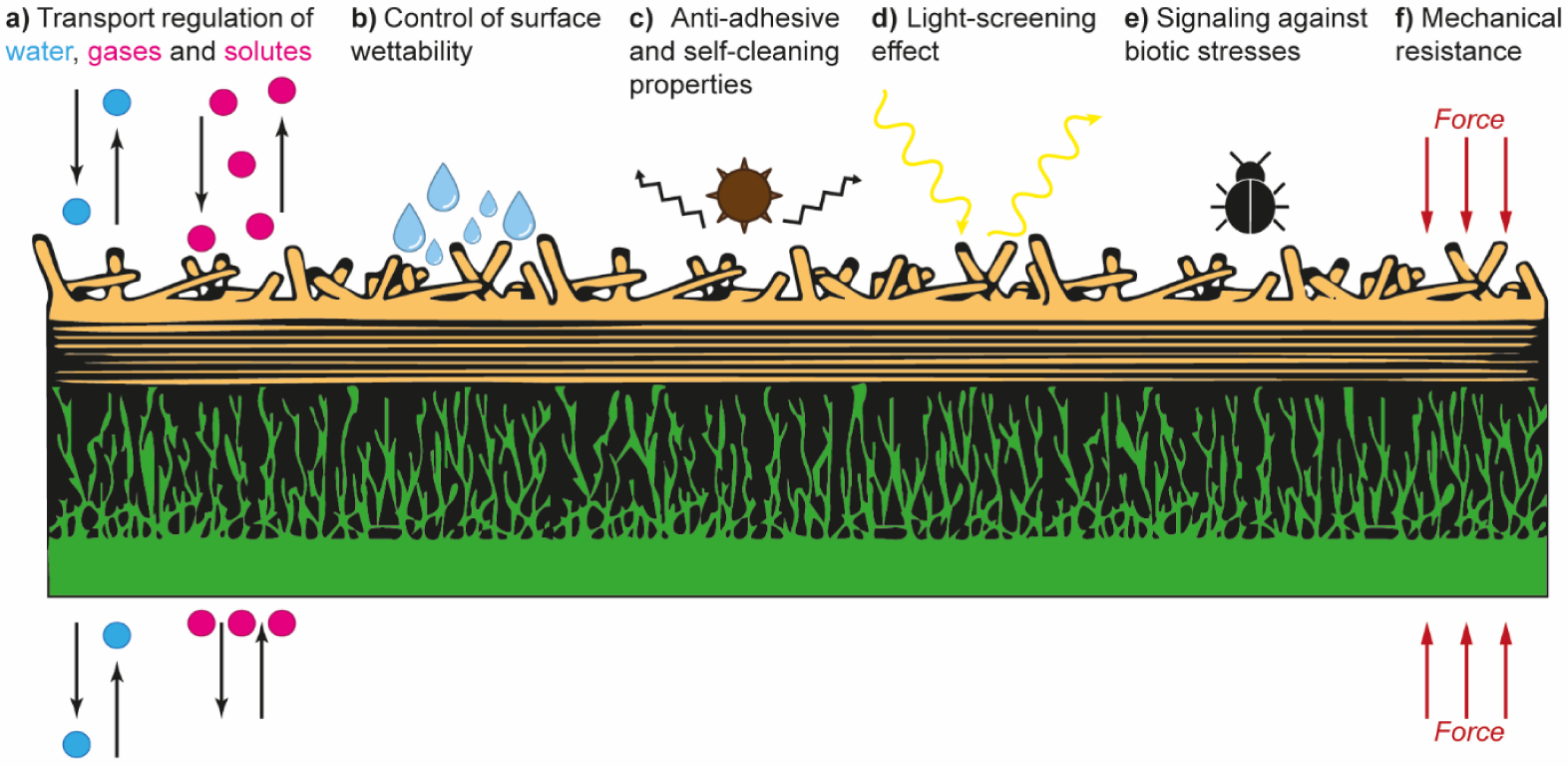
Schematic representation summarizing key functions of plant cuticles. (a) Regulation of bidirectional mass transport between the plant and its surroundings. (b) Modulation of surface wettability. (c) Antiadhesive and self-cleaning characteristics to reduce surface contamination and pathogen attacks. (d) Protection from UV radiation. (e) Coordination of plant-pathogen interactions with insects via signaling and regulation of epidermal cell differentiation and development. (f) Resistance against mechanical stress and preservation of the plant’s structural integrity. The schematic was redrawn based on ref 39.

Despite the early studies on directional water transport in insect cuticles discussed above,^29–31,33^ analogous investigations on the transport characteristics of plant cuticles were not reported until 1959.^63^ After isolating the cuticular membrane of ivy leaves, specifically from *Hedera helix* species, Schieferstein and Loomis measured the water permeability in two opposite transport directions (i.e., physiologically inward and outward directions), using a gravimetric method in which water was placed on one side of the cuticle, and the weight loss was monitored.^63^ As previously observed for insect cuticles, the water loss was greater in the inward direction, with an asymmetry factor *AF* (which is defined as the ratio of the permeabilities along the inward and the outward direction) ranging between 1.30 and 1.58, depending on the age of the leaf.^63^ Referring to Hurst’s study,^30^ the authors explained these results with molecular pores in the surface membrane that close upon dehydration when the direction of transport is outward.^63^

Beyond directional water transport, further evidence for asymmetric diffusion through cuticular membranes extracted from plants was presented in 1964, when Yamada et al. reported asymmetric penetration of ions in the cuticles isolated from tomato (*Lycopersicon esculentum*) fruits and green onion (*Allium cepa*) leaves.^64^ These two types of cuticular membranes were selected as model systems, representing stomata-containing (green onion leaf) and stomata-free (tomato fruit) cuticles, to investigate mechanisms of nutrient absorption and leaching. In line with the observations reported by Schieferstein and Loomis for water transport,^63^ the penetration rate of both cations and anions was consistently higher in the physiologically inward direction, irrespective of whether the cuticle was stomatous or astomatous.^64^ The authors attributed this asymmetric penetration to differences in the ion-binding capacities of the two cuticle surfaces, which facilitate the uptake of mineral nutrients.^64^

Support for asymmetric transport in plant cuticles was also provided in 1988 by Schönherr and Riederer, who studied the simultaneous bilateral desorption of the radiolabeled pesticide ^14^C-(2,4-dichlorophenoxy)­acetic acid in cuticular membranes isolated from four different plant species, i.e., *Citrus*, *Ficus*, *Lycopersicon*, and *Capsicum* (Figure 3).^65^ Investigating the fate of this chemical pollutant once sorbed in the plant cuticles, the authors reported remarkably higher desorption rates from the inner surface of all the examined cuticular membranes, with pollutant release proceeding 50–80-fold more rapidly than from the outer surface.^65^ Asymmetric desorption persisted even when the soluble cuticular lipids (waxes) present on the cuticles’ surface were removed by chloroform extraction, although the asymmetry factors within the residual polymer matrices were reduced to values between 6 and 7.^65^ In the case of a homogeneous membrane, simultaneous desorption from both surfaces should yield identical desorption curves, each asymptotically approaching a value of *M*_t_ */M*_∞_ = 0.5, if the release was symmetric and each of the two sides would release half of the pesticide. However, this theoretical behavior was not observed for either the wax-containing (CM) or the wax-free (MX) cuticular membranes isolated from citrus (*Citrus aurantium* L.) and pepper (*Capsicum annuum* L.), as evidenced by the desorption curves shown in Figure 3.^65^

**Figure 3:**
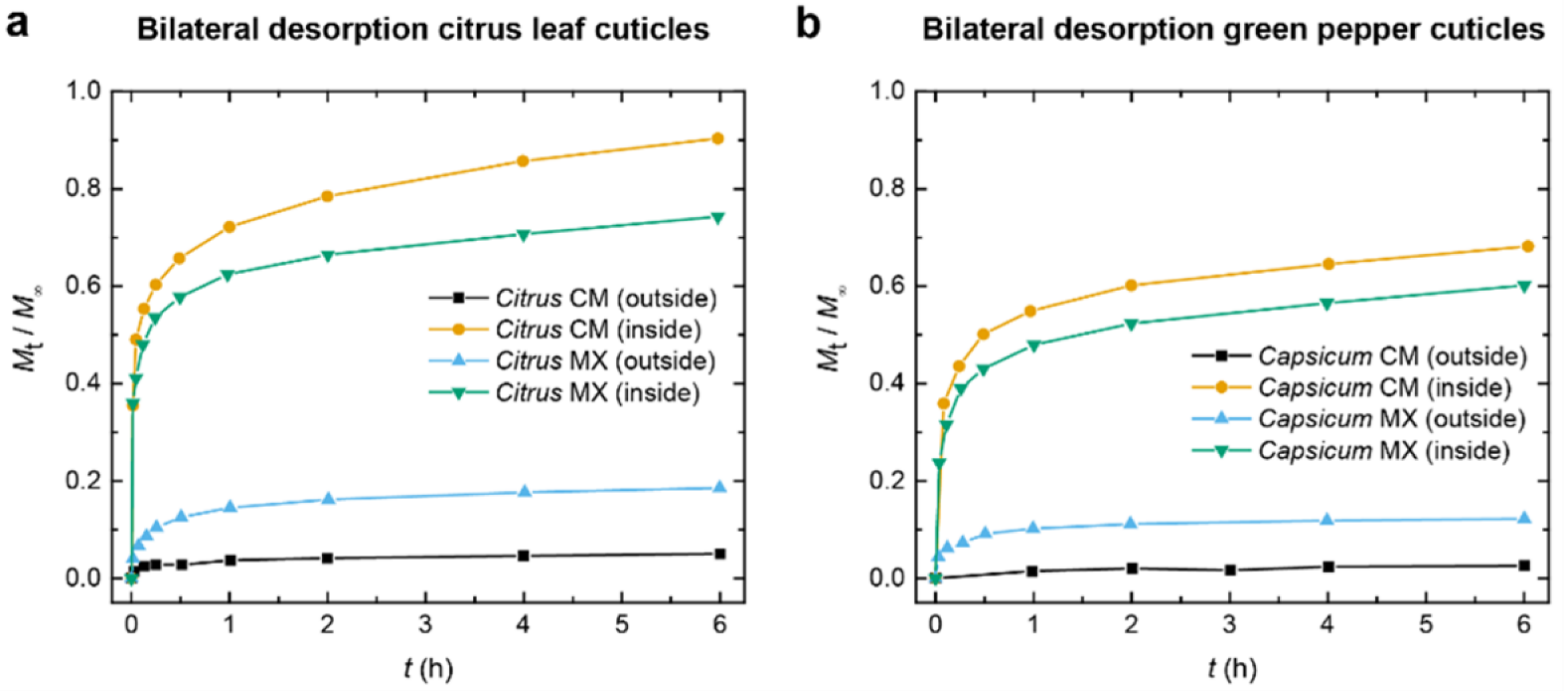
Bilateral desorption profiles of radiolabeled ^14^C-(2,4-dichlorophenoxy)­acetic acid from intact wax-containing (CM) and dewaxed (MX) cuticles isolated from (a) citrus (*Citrus aurantium* L.) leaves and (b) green pepper (*Capsicum annuum* L.) fruits. The graphs show data from ref 65 that were replotted.

Based on these findings, Schönherr and Riederer concluded that the observed asymmetrical desorption originated from structural heterogeneities within the plant cuticles.^65^ They considered that the epicuticular waxes play a major, though not essential, role in determining the asymmetric transport characteristics. The study presented evidence of a correlation between asymmetric transport and a heterogeneous architecture of plant cuticles, a hypothesis supported by the then-recent discoveries and observations of the internal graded structure of these biological membranes, made possible by the application of transmission electron microscopy (TEM) in the early 1980s.^38,44,45^

Following these initial investigations, the phenomenon of directional transport across plant cuticles remained remarkably unexplored, especially in the context of asymmetric water permeation.^40,64–67^ Eventually, Kamtsikakis et al. investigated the transport of tritiated water (^3^H_2_O) through astomatous cuticles extracted from leaves of olive (*Olea europaea*) or common ivy (*Hedera helix*) plants.^68^ The results obtained from intact (wax-containing) leaf cuticles of *Hedera helix* are presented in Figure 4 for transport in the physiological outward (Figure 4a) and inward (Figure 4b) direction.^68^ To probe how the hydration status affects the transport behavior of the cuticular membranes, experiments were conducted for two different sets of conditions, i.e., with the relative humidity of the receiver compartment (*RH*_R_) set to 2 or 100%, while relative humidity in the donor compartment (*RH*_D_) was kept at 100%. The data shows that for the outward direction, the ^3^H_2_O permeance rose by ∼33% as *RH*_R_ was changed from 2 to 100%, whereas changes in *RH*_R_ did not affect the permeance for the inward transport (Figure 4c).^68^

**Figure 4:**
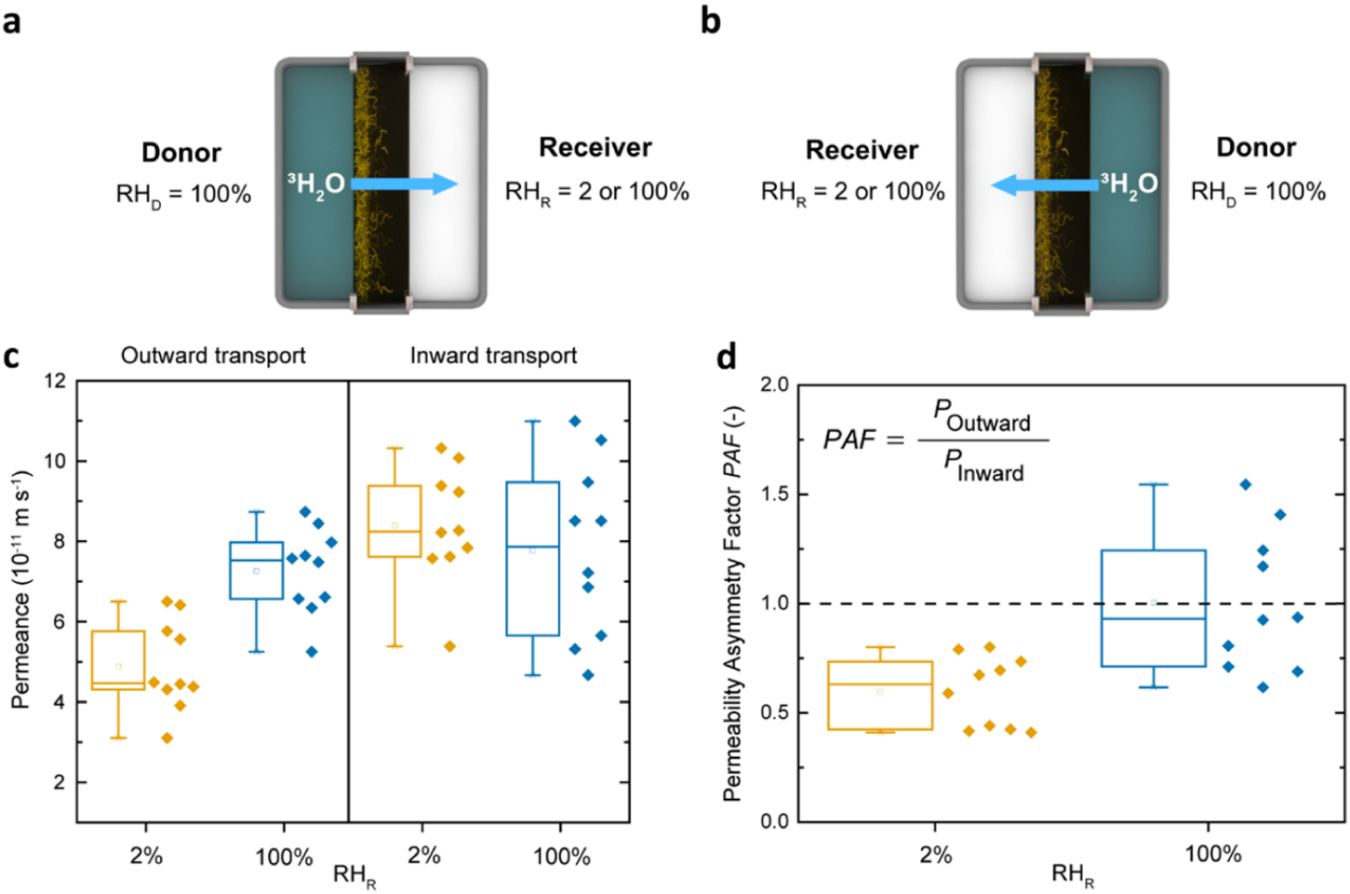
Schematic illustrations of (a) outward and (b) inward transport of radiolabeled water (^3^H_2_O) through ivy (*Hedera helix*) wax-containing leaf cuticles. Outward and inward transport indicate that either the inner or the outer cuticular side faces the donor compartment, which is maintained at a relative humidity *RH*_D_ = 100%. (c) ^3^H_2_O permeances measured in the two transport directions under different receiver relative humidity conditions (*RH*_R_ = 2 or 100%). (d) Permeability asymmetry factor (*PAF*), analogous to the asymmetry factor (*AF*), calculated from the ratio of outward and inward transport. Reproduced from ref 68. Available under a CC-BY license. Copyright 2021 Aristotelis Kamtsikakis, Johanna Baales, Viktoria V. Zeisler-Diehl, Dimitri Vanhecke, Justin O. Zoppe, Lukas Schreiber, Christoph Weder.

To quantify the extent of asymmetric transport through plant cuticles, the authors calculated the permeability asymmetry factor (*PAF*), also referred to as asymmetry factor (*AF*), by taking the ratio of outward to inward permeance, and observed a switchable directionality of transport.^68^ When the cuticular membranes were fully hydrated (*RH*_R_ = *RH*_D_ = 100%), the water transport was symmetric, as indicated by a *PAF* value close to unity (*PAF* = 1.01). In contrast, under a dry receiver condition (*RH*_R_ = 2%), water transport became asymmetric (*PAF* = 0.60), with inward exceeding outward transport (*PAF* < 1) (Figure 4d).^68^ These observations align with the report by Schieferstein and Loomis, who also observed a preferential water transport from the external environment toward the leaf interior when studying the water permeance of cuticles isolated from *H. helix*.^63^

Kamtsikakis et al. further reported similar findings for water transport through cuticular membranes isolated from *Olea europaea* leaves^68^ and investigated how it is affected by cuticular waxes. In line with the earlier findings of Schönherr and Riederer, who previously studied how various chemicals desorb from plant cuticles,^65^ the asymmetric transport under dry receiver conditions (*RH*_R_ = 2%), indicated by *PAF* < 1, persisted even after extraction of the cuticular waxes (Figure 5).^68^ More specifically, the authors reported that the *PAF* value decreased from 0.62 to 0.37 after wax removal, suggesting that the magnitude of directionality was further enhanced in the absence of cuticular waxes.^68^ Together with the observed increase in the outward ^3^H_2_O permeance upon increasing *RH*_R_ from 2 to 100%, these results led Kamtsikakis et al. to conclude that asymmetric water transport in plant cuticles is predominantly regulated by water uptake of the outer cuticular side rich in cutin, rather than by the inner polysaccharide side, an insight with significant ecological relevance.^68^ Under external arid conditions, the outward transport through the cuticular membrane is lower than the inward, and this asymmetric permeation contributes to the conservation of water within the plant. By contrast, during fog or rainfall events, the dense cutin layer of the outer cuticular side undergoes swelling and plasticization, which enhances inward transport and results in symmetric water transport through the plant cuticle. This humidity-dependent switching of water transport directionality represents an adaptive mechanism that enables the plant to either release excess water or to absorb atmospheric moisture, depending on its internal water balance.^68^

**Figure 5:**
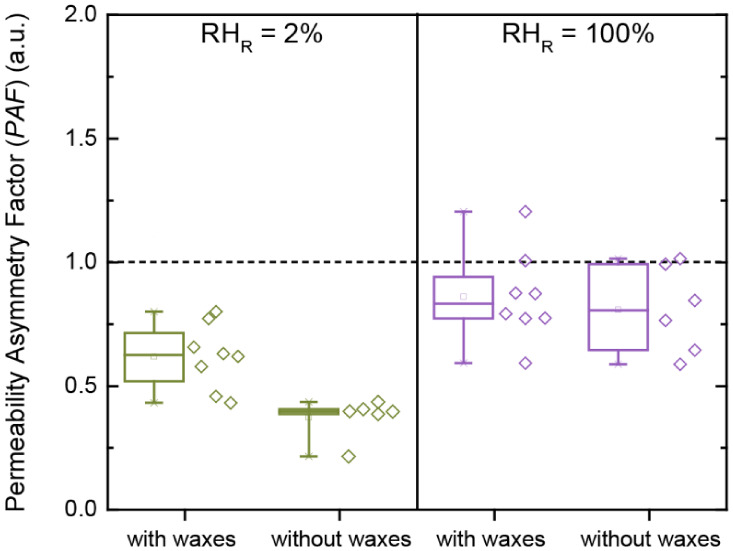
Permeability asymmetry factor (*PAF*), analogous to the asymmetry factor (*AF*), through olive (*Olea europaea*) cuticles with or without cuticular waxes under different conditions of relative humidity at the receiver compartment (*RH*_R_ = 2 or 100%). Reproduced from ref 68. Available under a CC-BY license. Copyright 2021 Aristotelis Kamtsikakis, Johanna Baales, Viktoria V. Zeisler-Diehl, Dimitri Vanhecke, Justin O. Zoppe, Lukas Schreiber, Christoph Weder.

Taken together, these studies on cuticles highlight that asymmetric water transport in biological membranes is intimately linked to structural and compositional gradients, as well as to the humidity-responsive permeability of specific membrane domains.^29–31,33,63,68^ Yet, despite the earlier evidence for directional permeation in insect cuticles,^29–31,33^ these biological systems were not considered in the first theoretical analyses of the phenomenon. In their pioneering studies on dense artificial composite membranes, Rogers et al. formulated the theoretical basis for asymmetric permeation without reference to earlier observations on insect cuticles.^32^ Although Petropoulos later cited insect cuticles as natural examples of membranes exhibiting directional water transport, the mechanistic basis of this behavior was not elaborated.^69^ Indeed, only Kamtsikakis et al. articulated in a recent study a clear connection between these theoretical works and the behavior of olive and ivy leaf cuticles, demonstrating that the water-induced plasticization of cutin,^60,70–73^ along with the compositionally graded structure of the cuticles, leads to asymmetric water transport through these biological membranes.^68^ Thus, plant cuticles satisfy the key conditions for directional permeation identified by Rogers et al. and function according to the theoretical framework detailed below.^32^

## 2 Theoretical Treatment of Mass Transport through Dense Asymmetric Polymer Membranes

The mass transport through dense nonporous polymer membranes can follow two main processes.^74^ They include capillary flow, where gases and vapors pass through microscopic cracks or microchannels through the membrane, and permeation, a solution-diffusion phenomenon in which gas or vapor molecules permeate the inter/intramolecular free volume of the polymer. In a defect-free, dense membrane, the primary mechanism for gas and vapor transport is permeation,^75^ which is treated here with a particular focus on the conditions required to achieve asymmetric permeation. For more details on transport by capillary flow, interested readers are referred to the works of Mahajan et al.,^76^ Rennie and Tavoularis,^77^ and González et al.^78^

### Solution-Diffusion Model

The mechanism of mass transport through a dense polymer membrane by way of permeation has been extensively studied, and it consists of three steps:^74,75,79,80^

(1) Permeant adsorption at the first membrane surface in accordance with Henry’s law, which is described by

1 c=Sp

where *c* is the sorbed permeant concentration at the first surface, *S* is its solubility coefficient in the polymer, and *p* represents the vapor pressure at equilibrium with the polymer.

(2) Permeant diffusion through the membrane in conformity with Fick’s first law:

2 $$J=-D\frac{\partial c}{\partial x}$$

where *J* is the area-normalized diffusive flux of the permeant through the membrane, $\frac{\partial c}{\partial x}$ describes its change in concentration along the transverse direction, and *D* is its diffusion coefficient.

(3) Permeant desorption at the second membrane surface, also in accordance with Henry’s law (eq 1).

Figure 6 shows a schematic of this solution-diffusion model. The first side of a one-dimensional membrane with thickness *L* is exposed to a generic permeant with a vapor pressure *p*_1_. The gas (or vapor) molecules dissolve at the interface and diffuse through the membrane driven by the concentration gradient *(c*_1_ *–c*_2_) across the membrane. The vapor pressure on the second side (*p*_2_) is thus lower than on the first side. Once a steady state is reached, the flux *J* no longer varies, allowing integration of eq 2 over the membrane thickness *L*. If the diffusion coefficient *D* does not vary with the permeant concentration, the integration of Fick’s first law results eq 3. For a rigorous, mathematical description of the integration steps, interested readers are invited to consult Robertson’s work.^74^

3 $$J=D\frac{\left(c_{1}-c_{2}\right)}{L}$$

**Figure 6:**
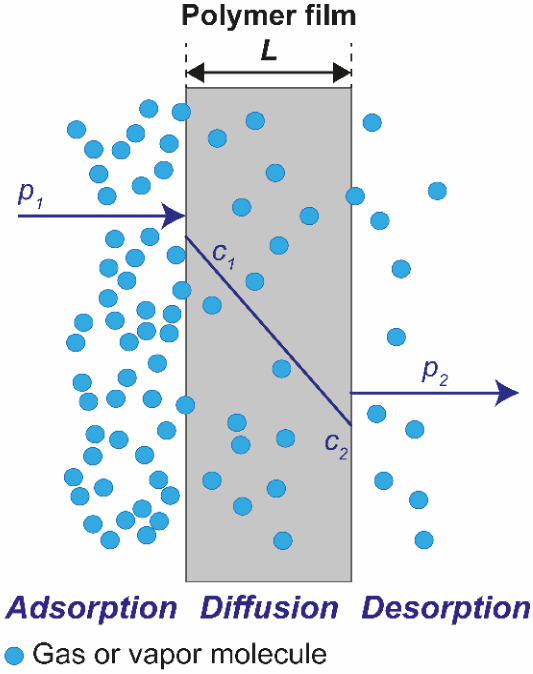
Schematic representation of gas/vapor permeation across a nonporous polymer membrane.

By applying Henry’s law (eq 1), the concentrations used in eq 3 are recast in terms of the vapor pressure of the permeant at the interface, affording eq 4 that expresses the permeant flux *J* across the membrane:

4 $$J=DS\frac{\left(p_{1}-p_{2}\right)}{L}$$

The permeability coefficient *P* is the product of *D* and the solubility coefficient *S*:

5 P=DS

and thus combines kinetic (*D*) and thermodynamic (*S*) terms.^74^

The permeant flux *J* can be expressed (and measured) by the permeant amount (*q*) that passes through a membrane with the surface area *A* during time *t*:^74,81^

6 $$J=\frac{q}{At}$$

By combining the expressions of the flux *J* given in eq 6 and 4 and using eq 5, *P* can be derived from experimentally accessible parameters:

7 $$P=\frac{qL}{At\left(p_{1}-p_{2}\right)}$$

The permeant quantity *q* can be expressed in mass, volume, or molar units. While volume units are often employed for gases such as O_2_ and CO_2_, mass units are typically used for the transport of water vapor. Despite these general considerations, over 30 different units for *P* appear in the scientific literature.^74,82^ An overview of the different units used to report *P* has been provided by Huglin and Zakaria.^82^ To simplify the comparison between different systems, all water permeability (*WP*) values reported in this review have been converted to the SI units kg m m^–2^ s^–1^ Pa^–1^.

Equation 5 is based on four main assumptions. First, diffusion occurs under steady-state conditions. Second, in-plane diffusion is absent, i.e., the diffusion of the permeant occurs only in the direction transversal to the membrane’s surface. Third, the relationship between the permeant concentration and the spatial coordinate through the polymer is linear. Fourth, the permeant concentration neither affects *D* nor *S*, which means that *P* is also concentration-independent. This assumption is valid for molecular diffusion processes that follow a Fickian behavior.^75,81^ However, if considerable permeant-polymer matrix interactions are in play, *D* and *S* are, in fact, concentration-dependent, and the diffusion process becomes non-Fickian. This behavior can be observed in glassy or semicrystalline polymers when the permeant species causes extensive swelling and thereby plasticization of the polymer,^75,81,83^ such as in hydrophilic polymers that are exposed to water.^74,75,81^ eq 5 shows that in case of a dependence of *D* and *S* on the permeant concentration, *P* must also be concentration-dependent. This dependence, which arises when specific permeant-polymer interactions are at play, is fundamental to achieving directional mass transport.

### Permeation through Laminated Systems

The expression of the permeability coefficient *P* given in eq 7 is valid for membranes that are compositionally homogeneous in the direction of flux, but it is of course possible to create gradients or combine multiple materials in a laminated structure to achieve specific transport characteristics that cannot be obtained using single-layer membranes. Multilayer polymeric membranes find applications in several fields, for example, energy production and gas separation,^84,85^ drug delivery and wound dressings,^86,87^ water treatment and filtration,^88^ and packaging.^89–91^

Considering a bilayer membrane as sketched in Figure 7, a first material with thickness *x*_1_ and permeability coefficient *P*_1_ is combined with a second material with thickness *x*_2_ and permeability coefficient *P*_2_. For the ideal case, the permeability coefficients of the two materials are constant and independent of the permeant concentration. The total membrane thickness *L* is given by the individual components, as reported in eq 8, and both layers are assumed to have the same cross-sectional area *A*, as expressed by eq 9:

8 $$L=x_{1}+x_{2}$$

9 $$A=A_{1}=A_{2}$$

**Figure 7:**
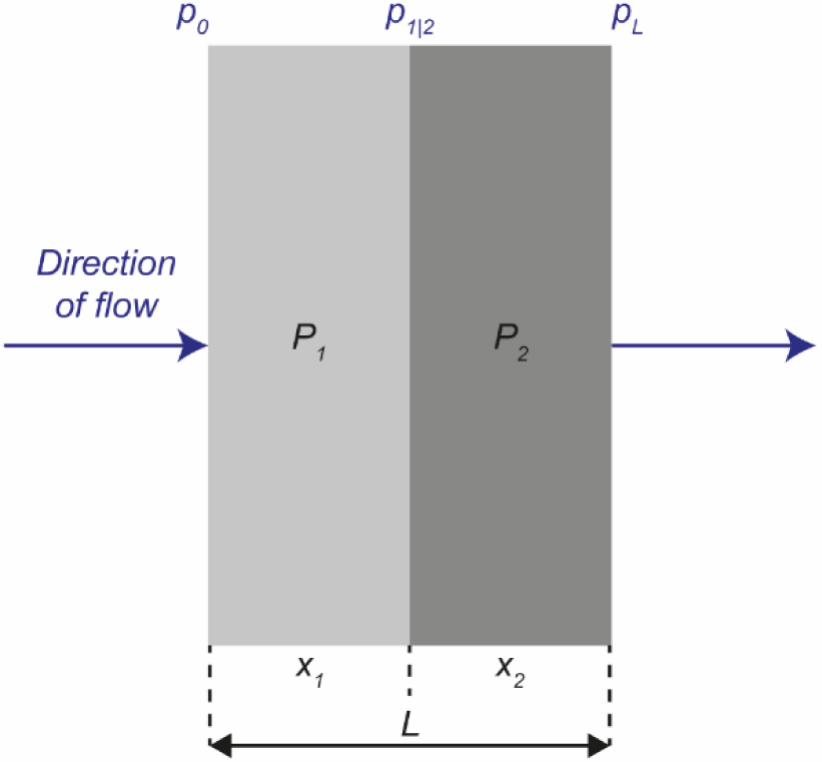
Schematic representation of permeation through a bilayer membrane composed of a first layer of thickness *x*_1_ and permeability coefficient *P*_1_ and a second layer of thickness *x*_2_ and permeability coefficient *P*_2_. The bilayer has a total thickness *L* and is subjected to the partial pressures *p*_0_ and *p*_L_, while *p*_1|2_ represents the partial pressure at the boundary of the two layers.

Here, *A*_1_ and *A*_2_ refer to the cross-sectional areas of the two layers. The bilayer membrane experiences an initial vapor pressure *p*_0_ at the upstream side and a final vapor pressure *p*_L_ on the outlet side. At the interface between the two layers, the vapor pressure is equal to an intermediate value *p*_1|2_. Assuming steady-state flux without accumulation or mass generation, the permeant amount *q* passing through the two layers during the time interval *t* is the same:

10 $$q=q_{1}=q_{2}$$

Here, *q*_1_ and *q*_2_ are the amounts of permeant passing through the first and second layers, respectively. Rearranging eq 7, *q* can be expressed by

11 $$q=P_{T}\frac{\left(p_{0}-p_{L}\right)At}{L}$$

where *P*_T_ and *L* are the laminated membrane’s total permeability coefficient and thickness, respectively, and (*p*_0_ *– p*_L_) is the total gradient of vapor pressure applied to the membrane. By rearranging eq 11, (*p*_0_ *– p*_L_) can be expressed as

12 $$\left(p_{0}-p_{L}\right)=q\frac{L}{P_{T}At}$$

The total vapor pressure gradient (*p*_0_ *– p*_L_) can also be expressed by the vapor pressure gradients acting on the two individual layers:

13 $$\left(p_{0}-p_{L}\right)=\left(p_{0}-p_{1|2}\right)+\left(p_{1|2}-p_{L}\right)$$

Here, *(p*_0_ *– p*_1|2_ *)* and *(p*_1|2_ *– p*_L_ *)* are the vapor pressure gradients across the first and second layer, respectively. Equations 12 and 13 can be combined to

14 $$q\frac{L}{P_{T}At}=q_{1}\frac{x_{1}}{P_{1}A_{1}t}+q_{2}\frac{x_{2}}{P_{2}A_{2}t}$$

where *x*_1_ and *x*_2_ indicate the thickness and *P*_1_ and *P*_2_ the permeability coefficients of the first and second layer, respectively. Using eq 10, eq 14 can be rearranged to afford:

15 $$\frac{L}{P_{T}A}=\frac{x_{1}}{P_{1}A_{1}}+\frac{x_{2}}{P_{2}A_{2}}$$

Since the transport area is the same for both layers (see eq 9), the total permeability of the bilayer membrane *P*_T_ is

16 $$\frac{1}{P_{T}}=\frac{x_{1}}{L}\frac{1}{P_{1}}+\frac{x_{2}}{L}\frac{1}{P_{2}}$$

Thus, given the assumptions of concentration-independent permeability and steady-state flow, the overall permeability of a multilayer membrane is a thickness-weighted sum of the permeabilities of its constituent layers.^74,75,92^

Occasionally, instead of permeability, the resistance to mass transport *R* is employed:^84^

17 $$R=\frac{L}{PA}$$

and the bilayer membrane’s overall resistance *R*_T_ is given by

18 $$R_{T}=R_{1}+R_{2}$$

where *R*_1_ and *R*_2_ are the resistances to mass transport of the two layers. Describing transport through multilayer films in terms of resistance is advantageous because each layer can be treated as a resistor in series.^74^ Based on this formalism, the total membrane resistance is simply the sum of the resistances of its constituent layers.^84,93^ Owing to its simplicity, the resistance-in-series model,^93,94^ which is also referred to as ideal laminate theory (ILT),^75,95–97^ is commonly employed to express the transport through laminated and graded membranes,^93–99^ even if the underlying assumption that the permeability coefficient of all layers is independent of the permeant’s concentration is not always applicable. Indeed, if the permeability coefficient of at least one of the materials combined in a multilayer structure depends on the concentration of the permeant, the heterogeneous spatial distribution of the layers can give rise to directional transport.

### Directional Permeation through Dense Membranes

The sorption and diffusion properties of homogeneous, dense polymeric membranes are typically position-independent, which renders permeation through homogeneous films symmetric.^100^ However, the mass transport through heterogeneous polymeric membranes that exhibit spatial inhomogeneities is much more complex, since sorption and diffusion properties can vary with the position,^100^ thereby causing the membrane permeability to depend on the flow direction.^69^ Such directional mass transport, also referred to as “valve” ^32,101^ or “flow reversal” effect,^69,102,103^ implies a direction-dependent permeation rate for a permeant traversing the membrane. Although the flow direction, of course, must follow the permeant concentration gradient, the absolute magnitude of the permeation rate varies, depending on which membrane side is exposed to the permeant. The first mathematical explanation for such directional permeation was articulated in 1948 by Hartley, who suggested that a membrane’s permeability can be asymmetric if *D* varies with position and permeant concentration.^31^ However, the principles governing this phenomenon were only rigorously formulated in the late 1950s, when the conditions for directional mass transport through dense membranes were theoretically and experimentally established by Rogers and coworkers.^32,100,101,104^ Their theoretical treatment treated a heterogeneous membrane as a series of *m* distinct layers, each with its own thickness *x*_n_ and permeability coefficient *P*_n_ (Figure 8).^32^ In the case of transport through this membrane according to the ILT, each *P*_n_ is independent of the vapor pressure *p* of the permeant species (Figure 8a). The permeant’s vapor pressure changes across the various layers, varying between *p*_0_ at the upstream side and *p*_L_ on the outlet side. At each interface between layers, the vapor pressure is expressed by *p*_n|(n+1)_. Under steady-state conditions, the permeation rate *J* is the same throughout the entire membrane and across all of its individual layers.^32^ *J* can be expressed by eq 19, which recalls the expression of the diffusive flux given by eq 4:

19 $$J=P_{1}\frac{\left(p_{1|2}-p_{0|1}\right)}{x_{1}}=P_{2}\frac{\left(p_{2|3}-p_{1|2}\right)}{x_{2}}=\cdot \cdot \cdot =P_{n}\frac{\left(p_{n|(n+1)}-p_{(n-1)|n}\right)}{x_{n}}$$

**Figure 8:**
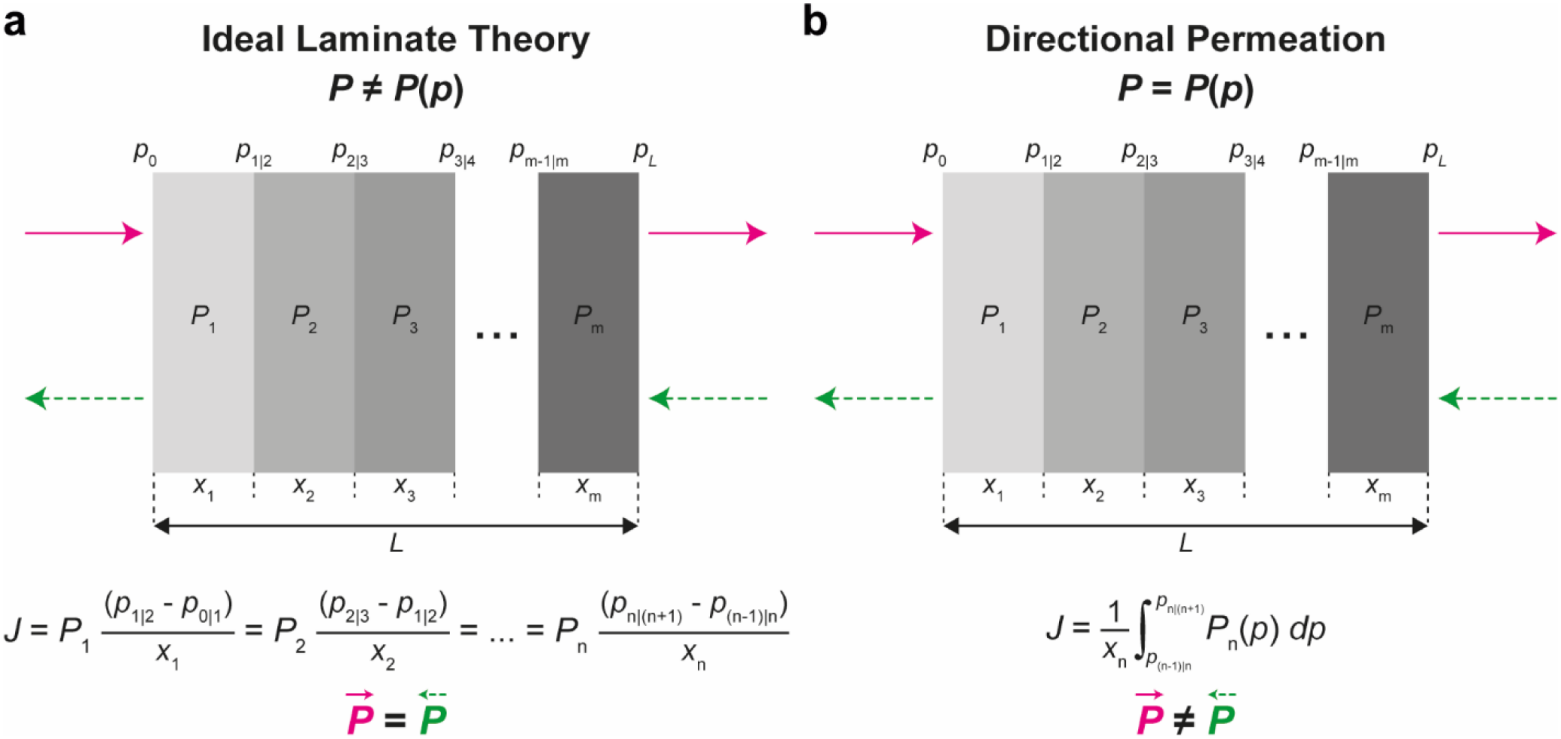
Schematic representation of permeation through dense multilayer membranes. (a) Transport according to the ILT. In this case, the individual layers have constant permeability coefficients (*P*) that do not change with vapor pressure (*p*), rendering permeation through the membrane, despite the heterogeneous structure, symmetric. (b) Directional permeation: asymmetric transport is expected in membranes where *P* varies with vapor pressure, and the permeability becomes position-dependent.

As discussed above, the assumption that the permeabilities of the membrane components do not vary with permeate concentration allows expressing the total permeability of the membrane (*P*_T_) as the thickness-weighted harmonic mean of the permeability coefficients of the individual layers (*P*_n_):^32^

20 $$\frac{1}{P_{T}}=\overset{m}{\underset{n=1}{\sum }}\frac{x_{n}}{L}\frac{1}{P_{n}}$$

Under such “ideal” conditions, the permeation rate across a heterogeneous multilayer membrane is independent of the layer order, resulting in symmetric transport behavior. However, if the permeability coefficients of the materials forming the multilayer membrane vary with the permeant’s vapor pressure (*P* = *P*(*p*), Figure 8b), which is position-dependent (*p* = *p*(*x*)), the expression for the permeation flow *J* given in eq 19 is no longer valid.^32^ Instead, the flow through a layer of thickness *x*_n_ with vapor-pressure-dependent permeability *P*_n_ = *P*_n_(*p*) is given by

21 $$J=\frac{1}{x_{n}}\int_{p_{(n-1)|n}}^{p_{n|(n+1)}}P_{n}\left(p\right)dp$$

and the permeation rate *J* becomes a function of *P*_n_(*p*). As a consequence, *P*_T_ changes when the layer order or flow direction changes. The *sine-qua-non* conditions for directional mass transport through dense membranes, which were first articulated by Rogers and co-workers, are thus: (1) spatial heterogeneity in the transverse direction, (2) at least one membrane layer whose permeability is a function of permeant vapor pressure, and (3) an external vapor-pressure gradient that induces plasticization of the permeant-sensitive layer.^32^

To experimentally validate this theoretical framework, Rogers et al. investigated a bilayer structure consisting of a polyamide 6 (PA6) and an ethyl cellulose film (Figure 9).^32^ On account of poor adhesion, the two polymer films did not adhere to each other and were thus merely placed in series. While the *WP* of polyamide 6 varies prominently with the water vapor pressure and increases by almost 1 order of magnitude when the applied relative humidity *RH* gradient rises from 0.24 to 0.98, the *WP* of ethyl cellulose is hardly affected (Figure 9a).^32^ The water-vapor-pressure dependence of the *WP* of PA6 is related to hydration, which causes the plasticization of the amorphous domains.^105^ In the bilayer structure, plasticization of the PA6 is more pronounced when the PA6 side faces a moist environment, and therefore the *WP* from this side can (at high RH values) be higher than in the opposite direction (Figure 9b). The magnitude of this asymmetry is expressed by the asymmetry factor *AF*, which for the PA6/ethyl cellulose bilayer membranes investigated increased from 1.5 at *ΔRH* = 0.19 to 3.4 at *ΔRH* = 0.96.^32^ The fact that *AF* depends on *ΔRH* highlights an important aspect, i.e., asymmetric transport can only be achieved under conditions where flipping the membrane changes the *WP* of the permeant-sensitive layer. In PA6/ethyl cellulose bilayer membranes, this is hardly the case when the humidity is too low to significantly plasticize PA6.

**Figure 9:**
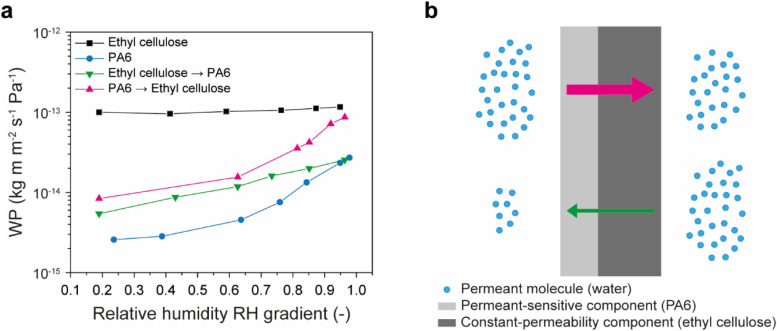
(a) Water permeability (*WP*) of polyamide 6 (PA6), ethyl cellulose, and a bilayer membrane assembled from these components in both transport directions as a function of the applied relative humidity (*RH*) gradient. The graph shows data from ref 32 that were replotted. (b) Schematic representation of the asymmetric transport through a bilayer membrane with a water-sensitive component (PA6). The water permeability of PA6 depends on the *RH* gradient, i.e., the water vapor pressure.

The required spatial heterogeneity in the transverse direction is achievable by changing the chemical composition, combining different components into an asymmetric architecture, or altering physical characteristics, such as the crystallinity or cross-link density, in single-component systems.^106^ Motivated by the biological and technological relevance of compositionally graded membranes for directional mass transport,^1^ different researchers sought to model these complex systems to predict and optimize the asymmetry factor theoretically.^69,107–110^ These studies examined the role of spatial heterogeneity in transport by assuming that the permeability coefficient depends intrinsically on both spatial position (*x*) and vapor pressure (*p*), such that *x* and *p* are nonseparable variables in the expression of *P­(x,p),* and several mathematical expressions of this relationship were considered.^69,107–109^ For example, Peterlin and Olf considered exponential and power functions for *P­(x,p)*, and demonstrated that an exponential expression leads to higher *AF* values.^107^

A more general mathematical expression of *P­(x,p)*, which includes the particular cases studied by Peterlin and coworkers,^107–109^ was later examined by Petropoulos, who remained, however, skeptical about its practical relevance for actual, realizable systems.^69,108^ Petropoulos also considered a compositionally graded membrane based on a series of graft copolymers of components A and B, in which the volume fraction of component A varies continuously across the membrane.^69^ Based on this new theoretical design, Petropoulos assumed that both components A and B exhibit a deviation from constant permeability and modeled how the continuous variation of volume fraction and the combination of the two permeability coefficients would affect the *AF*. This study suggests that the *AF* can be maximized by finely tuning intrinsic parameters, i.e., the permeability coefficients of the two components, as well as extrinsic factors, i.e., the gradual variation in composition and the vapor pressure gradient applied to the membrane.^69^

Petropoulos also conducted a theoretical study of laminated membranes, which suggests that the latter architecture is not less effective than graded structures with respect to the maximum *AF* that can be reached.^69^ Indeed, 20 years after Petropoulos’ work, Yamanaka provided the mathematical proof that, in a two-component membrane, under the assumption that only one component exhibits concentration-dependent permeability, the configuration in which *AF* is maximized is a laminated structure.^110^

Due to their simple geometry, multilayer membranes have been the focus of many theoretical studies aimed at modeling how such structures can be optimized to achieve maximum permeation contrast in the two transport directions.^22,69,102,110,111^ Most of these works have considered bilayer films with one layer (A) exhibiting a vapor-pressure-dependent permeability (*P*_A_ = *P*_A_(*p*)), whereas the second layer (B) was assumed to have a constant, concentration-independent permeability coefficient (*P*_B_ = const).^69,102,110,111^ Many of these studies clearly show that the relationship between permeability and permeant activity in component A (*P*_A_(*p*)) is crucial and must be included in the modeling framework.^69,102^ However, this is generally far from trivial,^102^ and the mathematical expressions of *P*_A_(*p*) adopted in these studies are often greatly simplified to obtain explicit formulas for the asymmetry factor *AF* in connection with tunable parameters. For example, in the study on the flow reversal effect in laminated membranes, Petropoulos considered the simple case of a step function in which *P*_A_ = *P*_0_ when the permeant activity is lower than a critical value, and *P*_A_ = *P*_a_ > *P*_0_ when the permeant activity exceeds this critical value.^102^ In addition to this simple stepwise change of *P*_A_(*p*), Petropoulos considered other mathematical expressions to correlate the concentration-dependent *P*_A_ and the permeant activity *p*, ranging from linear to exponential relationships or even inverse square functions.^69^ Depending on the assumed expression of *P*_A_(*p*), the asymmetry factor *AF* was reported to depend on both extrinsic parameters, such as the permeant activity applied at the upstream side of the laminated membrane and the thickness of the layers, as well as intrinsic properties of the components, e.g., the constant *P*_B_ value of the second component and the parameters underlying the mathematical correlation *P*_A_(*p*).^69,102^ Using this framework, Petropoulos demonstrated that the asymmetry factor can be optimized by properly tuning these parameters, and reported that *AF* values of nearly 10 should be theoretically achievable, but no realistic materials whose combination would give such high values were proposed.^69^

Despite extensive theoretical efforts to model directional transport in both graded and laminated membranes, only a few experimental studies were carried out to validate the predictions derived from mathematical models (*vide infra*).^67,103^ The reason for this lack of empirical investigations likely lies in the fact that most reported models rely on assumed mathematical correlations between the permeability coefficients and the permeant’s vapor pressure, without providing examples of real materials with the postulated concentration-dependent permeability.^69,102^ To simplify the theoretical treatment of mass transport equations, most studies have employed simple mathematical relationships between permeability and permeant activity, thereby diverging from the inherent complexity of real systems.

## 3 Directional Water Transport in Artificial Dense Membranes

### Concentration-Dependent Permeability in Moisture-Sensitive Materials

As discussed above, at least one membrane component must exhibit concentration-dependent permeability (*P*) to achieve directional mass transport. This condition is typically met due to deviations from classical Fickian transport, i.e., *D* and/or *S* vary with the concentration of the permeant species.^74,75^ Such non-Fickian behavior is observed in glassy or semicrystalline polymers if the permeant induces significant swelling of the polymer matrix.^75,81,83^ A representative example is the PA6 discussed above, where the changing permeability is related to the humidity-dependent glass transition temperature (*T*_g_). Dlubek et al. demonstrated that the *T*_g_ of PA6 decreases with increasing relative humidity, concomitant with an increase in free volume.^105^ Since enhanced free volume promotes molecular mobility and diffusion,^74,75^ this finding provides a physical rationale for the moisture-induced increase in *WP*. This behavior was comprehensively examined by Del Nobile and coworkers in the context of packaging applications.^112^ Employing a mechanistic approach, the authors decomposed the overall permeation process into two fundamental stages: water sorption (or solubilization) and diffusion. Sorption was described in terms of specific interactions of water with hydrophilic sites along the polymer molecules, and diffusion was considered non-Fickian by treating the diffusion coefficient as a function of local water concentration.^112^ The integration of these two contributions enabled the accurate prediction of the experimentally observed increase in *WP* with water activity.^112^ Building on this approach, Del Nobile et al. successfully extended their mathematical treatment to other hydrophilic polymers, including poly­(ethylene vinyl alcohol) (EVOH) copolymers and cellophane, confirming the general validity of their theoretical framework across different hydrophilic polymers.^113,114^

Among these materials, EVOH represents a particularly instructive case for the interplay between humidity and barrier performance in hydrophilic polymers.^114–117^ In an early investigation of the relation between water sorption and the *T*_g_ of EVOH, Zhang et al. reported that the *T*_g_ decreases progressively with increasing relative humidity (*RH*).^115^ The authors further demonstrated that the extent of *T*_g_ depression also depends on the copolymer composition (i.e., the ratio of ethylene to vinyl alcohol residues) and on the molecular orientation within the semicrystalline EVOH films.^115^ The authors subsequently investigated how the moisture-induced reduction in *T*_g_ impacts the barrier properties of EVOH,^116^ and demonstrated that both the water vapor transmission rate (*WVTR*) and the oxygen transmission rate (*OTR*) increase markedly with *RH*. This dependence was attributed to *T*_g_ depression and plasticization of the material, along with a concomitant increase in chain mobility and free volume, which facilitated the diffusion of small penetrant molecules through the polymer matrix.^115,116^

Poly­(vinyl alcohol) (PVA) represents another illustrative example of a polymer whose water permeability strongly depends on the *RH*.^103,118–121^ One of the earliest observations of this dependence was reported in 1948 by Hauser and McLaren, who examined water permeation through various polymeric materials and noted that PVA exhibits a pronounced sensitivity to the *RH*.^118^ Indeed, the PVA’s water permeability increased by nearly a factor of a thousand as the *RH* was increased from 0.55 to 1.0, following a sigmoidal trend when plotted on a semilogarithmic scale (Figure 10).^122^ Similar to PA6, this pronounced humidity dependence is associated with the plasticization of the polymer matrix upon swelling with water, which affects both the *T*_g_ and the free volume.^122–124^ In 1996, Hodge et al. demonstrated that water molecules absorbed by the amorphous domains of PVA act as molecular lubricants, disrupting hydrogen bonds among PVA chains.^123^ Through this mechanism, chain mobility and free volume increase, resulting in a progressive decrease in *T*_g_ with increasing water uptake.^122,123^

**Figure 10:**
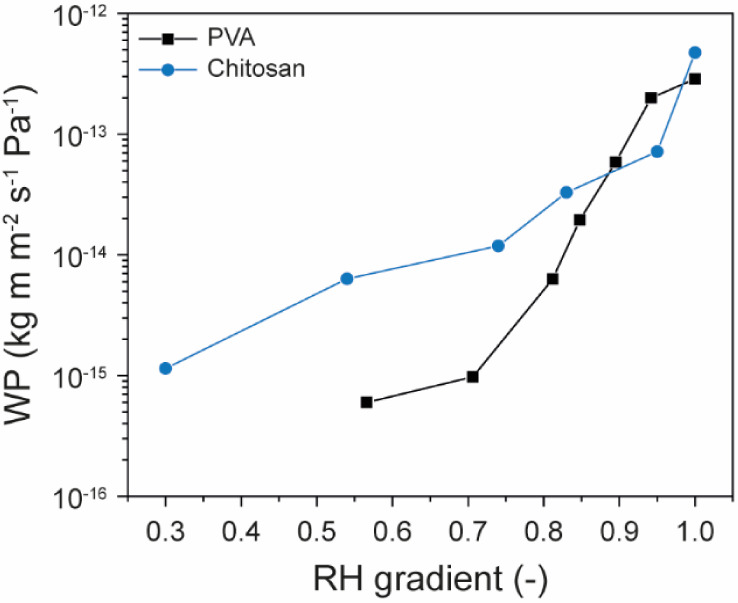
Water permeability (*WP*) of two moisture-sensitive materials: poly­(vinyl alcohol) (PVA) and chitosan. The graph shows data from ref 122 (PVA) and ref 125 (chitosan) that were replotted.

More recently, Hu et al. extended the understanding of moisture-induced plasticization in PVA by investigating how simultaneous exposure to moisture and heat affects the viscoelastic behavior via dynamic mechanical analysis (DMA).^124^ The results show a nearly linear decrease in the *T*_g_ of PVA with increasing *RH*, with values ranging from >70 °C at *RH* = 0% to <20 °C at *RH* = 60%.^124^ In addition to this trend, Hu et al. identified a glass-to-rubber transition in PVA under isothermal conditions, which was induced solely by the increase in *RH* during DMA measurements.^124^ They introduced the concept of glass transition relative humidity (*RH*_g_), defined as the critical humidity where the PVA transitions from a glassy to a rubbery material, and demonstrated that *RH*_g_ decreases linearly with increasing temperature.^124^

The dependence of *T*_g_ on *RH* provides a quantitative description of the plasticization mechanism in hydrophilic polymers such as PVA and constitutes a valuable indicator of potential moisture-dependent water permeability. In this context, Hu et al. reported the *RH*-dependent *T*_g_ of other polymers,^124^ including poly­(vinylpyrrolidone) (PVP)^126^ and hydroxypropyl methylcellulose phthalate (HPMCP),^127^ which both may serve as moisture-sensitive components in systems designed to achieve directional water transport.

Another commodity polymer exhibiting pronounced moisture sensitivity is chitosan, a biobased polysaccharide produced by the partial deacetylation of chitin, the primary component of the exoskeletons of crustaceans and arthropods.^125,128,129^ As a renewable and biodegradable material, chitosan has attracted considerable attention for sustainable packaging and membrane applications.^130–132^ In an early study, Despond et al. systematically investigated how *RH* affects the water-vapor and gas-barrier performance of chitosan films.^125^ The authors reported that the *WP* of chitosan rises by 2 orders of magnitude from 1.1 × 10^–15^ kg m m^–2^ s^–1^ Pa^–1^ to 4.7 × 10^–13^ kg m m^–2^ s^–1^ Pa^–1^ as the *RH* increases from 0.3 to 1.0 (Figure 10).^125^ Similar to the behavior reported for EVOH,^116,117^ concomitant increases in O_2_ and CO_2_ permeability were observed, revealing the strong plasticizing influence of water on this biobased polymer.^125^ These findings were further corroborated by Aguirre-Loredo et al., who established a direct correlation between the moisture-induced increase in *WP* and a reduction in the *T*_g_ of chitosan with increasing water activity.^129^ Although chitosan has not yet been used to achieve directional water permeation in dense membranes, its concentration-dependent *WP* makes it a promising biobased candidate for future asymmetric membrane configurations. In contrast, PA6, EVOH, and PVA, the other materials discussed in this section, have already been successfully employed in dense asymmetric membranes with directional water transport behavior (*vide infra*).

### From Cuticle-Inspired Membranes to Directional Water Transport: State of the Art

Interestingly, there are many examples of cuticle-inspired dense membranes in which the authors focused on replicating aspects of the plant cuticle barrier functions without investigating the directionality of the transport properties. For example, numerous authors have reported synthetic analogues of cutin,^133–139^ which represents the main component of these biological membranes (40–80% of the cuticle’s dry weight).^26,138^ One of the first attempts to synthesize cutin from its primary constituents was reported in 2004, when Benítez et al. performed the polycondensation of monomers isolated from tomato fruit cutin (*Lycopersicon esculentum*).^133^ A similar approach was investigated by Gómez-Patiño and colleagues, who synthesized aliphatic polyesters starting from 10,16-dihydroxyhexadecanoic acid (diHPA) (i.e., the main monomer of tomato cutin), which was obtained from the depolymerization of tomato cutin contained in agro-waste residuals.^139^

As an alternative to the use of monomers extracted from cutin, a plant-cutin-mimicking polymer was synthesized from aleuritic acid, whose polycondensation yielded a polyester closely resembling the natural component of cuticles.^134^ Heredia-Guerrero and coworkers employed the resulting polyaleuritate as a key component in the development of cuticle-inspired materials.^140,141^ More specifically, a mixture of carnauba wax and aleuritic acid was sprayed onto a fibrous cellulose substrate, which was then hot-pressed to form a polyaleuritate coating. The cuticle-like composite structure was considered for packaging applications.^141^ This sequential process of spray coating and hot-pressing was also employed to form bilayer membranes using cellulose nanocrystal (CNC) films as substrate (Figure 11).^140^ Without compromising the optical characteristics of the photonic CNC films, the polyaleuritate coating enhanced the mechanical and water-barrier properties, playing a role similar to that of the cutinized cell wall layer in plant cuticle and improving the performance of this functional packaging material.^140^

**Figure 11:**
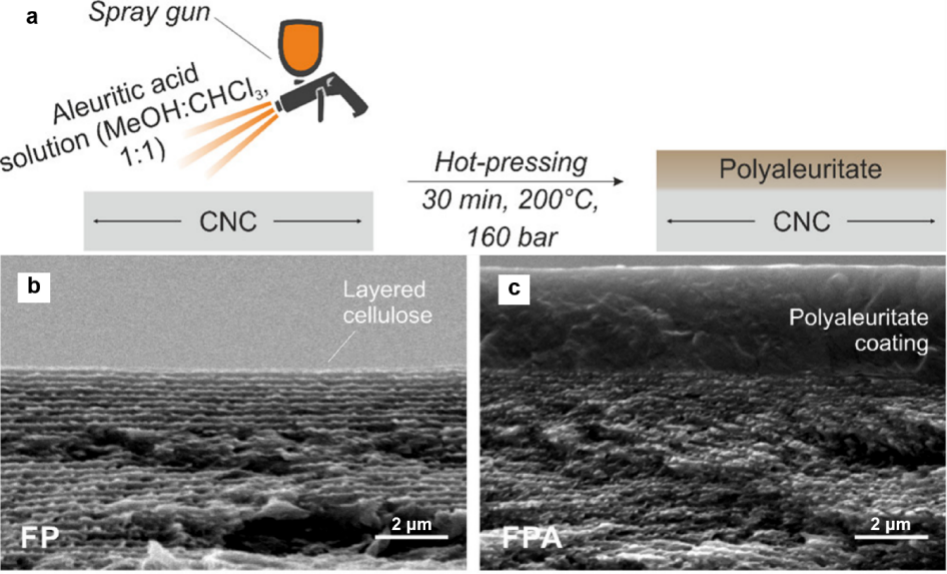
(a) Sequential process of spray coating and hot-pressing for the preparation of bilayer membranes using cellulose nanocrystal (CNC) films as substrate. Cross-sectional SEM images of a CNC film substrate (FP) (b) before and (c) after the coating with polyaleuritate, resulting in the bilayer membrane (FPA). Reproduced with permission from ref 140. Copyright 2020 American Chemical Society.

The fabrication of simplified bilayer structures to replicate the complex architecture of plant cuticles is a widely adopted strategy.^140,142–144^ In this approach, the bioinspired artificial membranes usually combine a relatively hydrophilic bottom layer, mimicking the inner cuticular side rich in polysaccharides, with a top hydrophobic layer that replaces the wax-coated, cutin-rich outer side. For example, Jullok and coworkers developed a cuticle-inspired membrane by combining a lower hydrophilic layer of poly­(phenylsulfone) (PPSU) with an upper layer of poly­(dimethylsiloxane) (PDMS).^143^ This bilayer membrane was employed in pervaporation processes for the recovery of aroma compounds from water.^143^ In another study, Zhang and Uyama reported a bilayer membrane in which a cellulose film (hydrophilic lower layer) was coated with hyperbranched poly­(ricinoleic acid) (HBPRA) (hydrophobic upper layer) to produce a transparent cuticle-mimicking film for use in packaging applications (Figure 12).^142^

**Figure 12:**
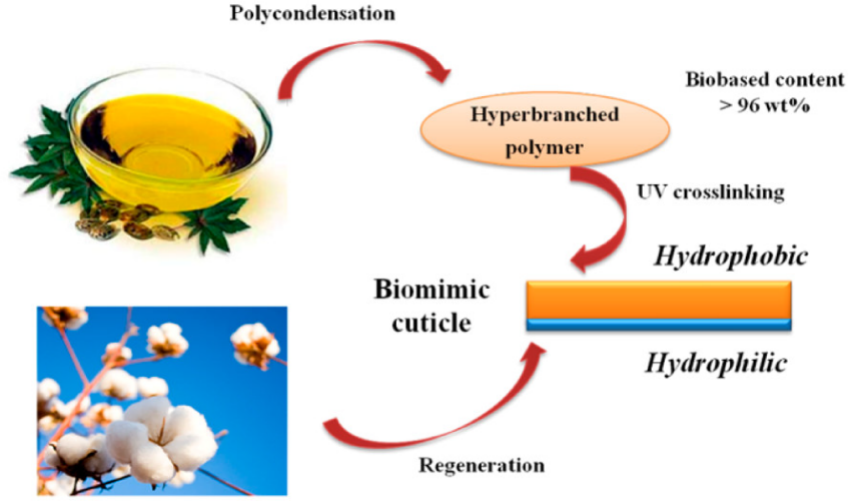
Cuticle-inspired bilayer membrane consisting of a hydrophilic cellulose substrate coated with a hydrophobic layer of hyperbranched poly­(ricinoleic acid). Reproduced with permission from ref 142. Copyright 2016 American Chemical Society.

More recently, Anusuyadevi and coworkers developed a laminated structure in which a CNC/glucose film was sandwiched between two cutin-like polyester films synthesized from 16-hydroxyhexadecanoic acid (HHA) monomers.^144^ Similarly to what was observed by Heredia-Guerrero et al.,^140^ the cuticle-inspired coating improved the water resistance of the CNC/glucose film while preserving its optical properties.^144^

In addition to the fabrication of laminated structures, several studies have also explored blending strategies to develop cuticle-inspired membranes, in which cutin or cutin-like materials are combined with polysaccharides and other components to primarily replicate the heterogeneous chemical composition of natural plant cuticles.^145–148^ For example, cutin extracted from tomato (*Solanum lycopersicum*) peels was mixed with pectin to produce hydrophobic edible films for food packaging applications.^145^ Similarly, Tedeschi and colleagues produced cuticle-inspired films by blending cutin-rich tomato pomace with sodium alginate and beeswax.^146^ Since the fabrication procedure involved only green solvents and heat to polymerize fatty acids derived from agro-waste, they reported this material as a sustainable alternative to traditional plastic for packaging applications.^146^ In 2021, two studies reported the use of cutin-like polyesters synthesized from HHA^147^ and diHPA^148^ monomers, mixed with glycerol, for the preparation of cuticle-inspired flexible packaging materials.

Drawing inspiration from the compositionally graded structure of olive leaf cuticles and specifically targeting asymmetric water transport, the Weder group combined the hydrophobic copolymer poly­(styrene)-*block*-poly­(butadiene)-*block*-poly­(styrene) (SBS) and hydrophilic CNCs to create directional nanocomposite membranes (Figure 13).^68^ To reproduce the asymmetric architecture found in the leaf cuticles (Figure 13a), a transversal CNC concentration gradient was produced by gravimetric sedimentation, affording membranes with a CNC-rich and a CNC-poor side (Figure 13b).^68^ The resulting compositionally graded membranes displayed asymmetric water permeation characteristics when a relative humidity gradient was applied, with a higher *WP* for water transport from the CNC-rich to the CNC-poor side (Figure 14).^68^ This behavior was attributed to the higher water affinity of the CNC-rich region, in agreement with the extensive literature on CNC–water interactions and humidity responsiveness,^149–152^ which would favor water uptake and facilitate permeation when this side faces the donor environment.^68^ However, the exact mechanism responsible for the directional permeation in this particular system remains unclear. With regard to the switchable character of the asymmetric water transport, a key feature previously observed in the olive leaf cuticles that inspired these SBS/CNC nanocomposites, the *PAF* of the artificial membranes changes little when the donor humidity (*RH*_D_) is varied between 75 and 100% with a receiver humidity (*RH*_R_) of 0% (Figure 14a). However, the permeation tests conducted with radiolabeled water ^3^H_2_O showed that the *PAF* of the SBS/CNC nanocomposites reduces to unity when both sides are hydrated, i.e., *RH*_R_ = *RH*_D_ = 100% (Figure 14b), in close analogy to the response observed in the olive leaf cuticles (Figure 5).^68^

**Figure 13:**
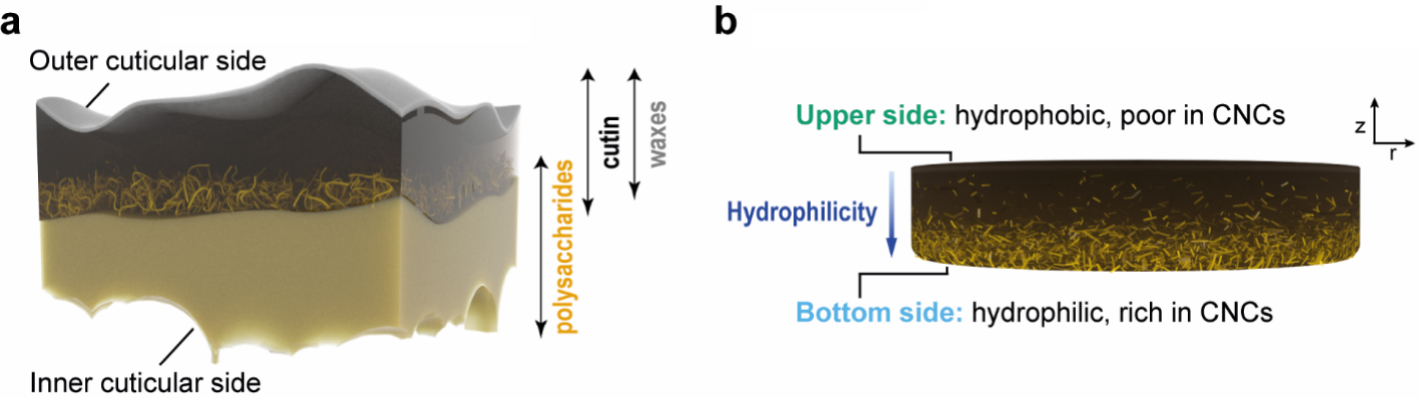
Schematic representations of the gradient structures in (a) olive leaf cuticles and (b) SBS/CNC nanocomposite membranes inspired by such cuticles. Reproduced from ref 68. Available under a CC-BY license. Copyright 2021 Aristotelis Kamtsikakis, Johanna Baales, Viktoria V. Zeisler-Diehl, Dimitri Vanhecke, Justin O. Zoppe, Lukas Schreiber, Christoph Weder.

**Figure 14:**
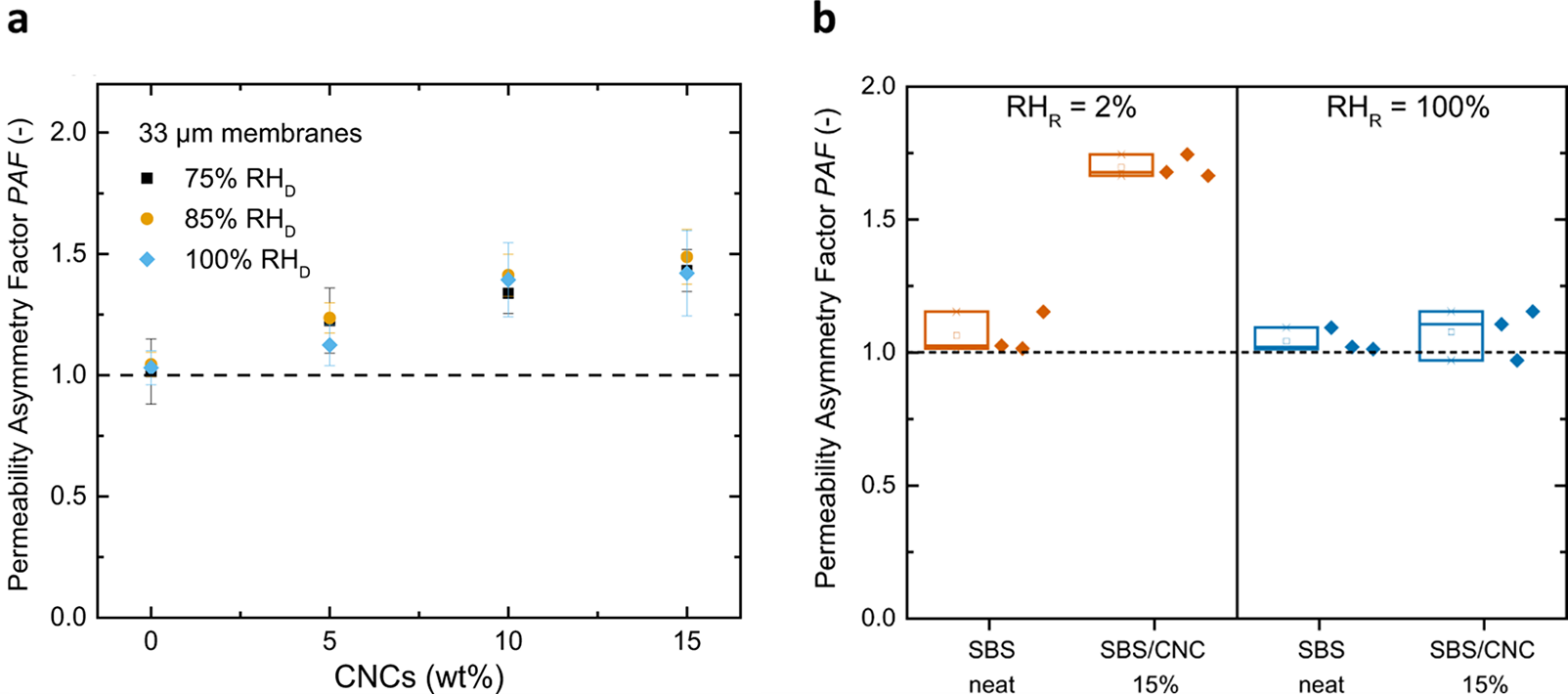
(a) Permeability asymmetry factor (*PAF*, identical to the asymmetry factor *AF*), of SBS/CNC nanocomposite membranes as a function of the relative humidity on the donor side (*RH*_D_) and the CNC content. The relative humidity on the receiver side (*RH*_R_) was ca. 0%. (b) *PAF* of SBS or SBS/CNC membranes measured by radiolabeled water ^3^H_2_O permeation tests, where the CNC-rich and the CNC-poor sides were facing the donor at *RH*_D_ = 100% and *RH*_R_ was 2 or 100%. Reproduced from ref 68 Available under a CC-BY license. Copyright 2021 Aristotelis Kamtsikakis, Johanna Baales, Viktoria V. Zeisler-Diehl, Dimitri Vanhecke, Justin O. Zoppe, Lukas Schreiber, Christoph Weder.

In a follow-up study, the authors reported asymmetry factors of up to 3 for SBS/CNC membranes containing 15% CNCs, whereas functionalizing the CNCs with nonpolar oleic acid groups (OLA-CNCs) decreases the *PAF* to ca. 1.2 in both water and ethanol.^153^ This effect was linked to a reduction in accessible sorption sites on the CNC surfaces coupled with improved OLA-CNC dispersion in the SBS matrix, resulting in a less pronounced compositional gradient.^153^

These SBS/CNC nanocomposites represent the first reported example of cuticle-inspired membranes displaying asymmetric water permeation, though not the first artificial membranes to exhibit this behavior.^68^ With the goal of providing a comprehensive review of the state of directional permeation, and focusing exclusively on water transport, we summarize pertinent studies in this section. Table 1 highlights the architecture and materials, fabrication method, preferential transport direction, and maximum asymmetry factor (*AF*) of dense membranes for which directional water transport has been reported.

**Table 1:** Overview of Artificial Dense Membranes with Directional Water Transport Characteristics

Membrane’s structure and components	Fabrication procedure	Preferential direction of transport	Max Asymmetry factor (*AF*)	Year	Ref
**Graded membranes:** gradual change in composition in the transverse direction					
Graded membranes based on poly(ethylene) (PE) and PVA grafts (graft concentration varies along the transverse direction)	Directed diffusion of vinyl acetate (VAc) into PE membrane, radiation-induced polymerization, and hydrolysis of PVAc to PVA	PVA → PE	6.5	1965	^104^
Graded membranes based on PE homogeneously and quaternized poly(2-vinylpyridine) (2VP) grafts (degree of quaternization varies along the transverse direction)	Radiation-induced grafting of 2VP on PE membrane followed by directed exposure to methyl bromide vapors, which cause quaternization of VP	Quaternization direction	2.0	1968	^154^
Graded membranes based on quaternized poly(styrene-*alt*-4-vinylpyridine) (degree of quaternization varies along the transverse direction)	Directed exposure to methyl bromide vapors, which cause quaternization of VP	Quaternization direction	1.6	1971	^155^
Graded membranes based on oxidized poly(l-methionine) (PLM) (degree of oxidation varies along the transverse direction)	Directed exposure to hydrogen peroxide, which causes oxidation of PLM	Oxidation direction	1.5	1978	^156^
Graded membranes based on partially hydrolyzed poly(ethylene-*co*-vinyl acetate) (EVA) (degree of hydrolysis varies along the transverse direction)	Directed exposure to alkaline solution, which causes hydrolysis of EVA	Hydrolysis direction	2.5	2005	^157^
Graded poly(styrene)-*block*-poly(butadiene)-*block*-poly(styrene) (SBS)/Cellulose nanocrystal (CNC) nanocomposite membranes (CNC concentration varies along the transverse direction)	Solvent casting process in which the colloidally unstable CNCs sediment during solvent evaporation	CNC-rich → CNC-poor	3.0	2021	^68,153^
Graded SBS/oleic acid-modified cellulose nanocrystals (OLA-CNCs) nanocomposite membranes (OLA-CNC concentration varies along the transverse direction)	Solvent casting process in which the colloidally unstable OLA-CNCs sediment during solvent evaporation	CNC-rich → CNC-poor	1.2	2021	^153^
Graded SBS/PVA nanofiber nanocomposite membranes (PVA concentration varies along the transverse direction)	Solvent casting of SBS solution on a porous mat of electrospun PVA nanofibers	PVA → SBS	2.3	2024	^158^
**Laminated membranes:** abrupt change in composition in the transverse direction					
2-Layer laminates of polyamide 6 (PA6) and ethyl cellulose	Two individual films mounted in series	PA6 → Ethyl cellulose	3.4	1957	^32^
2-Layer laminates of poly(chloroprene) (CR) and ethylene-propylene-diene terpolymer (EPDM)	Molding of individual layers and subsequent lamination	EPDM → CR	1.2	1983	^159^
2-Layer laminates of styrene–butadiene rubber (SBR) and EPDM	Molding of individual layers and subsequent lamination	EPDM → SBR	1.8	1986	^160^
2-Layer laminates of EPDM and nitrile butadiene rubber (NBR)	Molding of individual layers and subsequent lamination	EPDM → NBR	1.6	1986	^161^
2-Layer laminates of poly(vinyl alcohol) (PVA) and poly(vinyl acetate) (PVAc)	Lamination by pressing preformed films	PVA → PVAc	1.6	1987	^103^
2-Layer laminates of PVA and poly(ethylene terephthalate) (PET)	Lamination by casting PVA solutions onto PET films	PVA → PET	4.0	1987	^103^
2-Layer laminates of PVA and SBS	Lamination by casting SBS solutions onto PVA film	PVA → SBS	5.8	2025	^122^
2-Layer and 3-layer laminates of a terpolymer (TP) of poly(HEMA-*co*-HEA-*co*-EHMA), SBS, and SBS-TP blends	Lamination by compression molding of preformed SBS, SBS-TP blend, and TP films	TP → SBS	4.0	2025	^162^
Laminated membranes of PVA and glycol-modified poly(ethylene terephthalate) (PETG)	Lamination by compression molding of preformed PVA and PETG films combined with a reactive adhesive interlayer	PVA → PETG	6.7	2026	^163^

Another approach to creating asymmetric, dense membranes involves applying directional chemical treatments to initially homogeneous membranes. Among these are compositionally graded membranes made by the directional quaternization of poly­(ethylene-*graft*-2-vinylpyridine)^154^ and poly­(styrene-*alt*-4-vinylpyridine) membranes.^155^ In both cases, the reaction mixture used to achieve quaternization was applied asymmetrically, i.e., to only one side of the membrane. The compositional gradients thus created lead to anisotropic hydration profiles, resulting in directional water transport with *AF* = 1.6–2 and water flux along the quaternization gradient exceeding that in the opposite direction (Table 1).^155^ A similar approach was taken by Minoura and coworkers, who exposed one side of a preformed poly­(l-methionine) (PLM) film to aqueous hydrogen peroxide.^156^ The resulting asymmetric oxidation of methionine to methionine sulfoxide generated a compositional gradient across the membrane and increased water sorption, collectively inducing higher water permeation from the oxidized surface toward the untreated side, albeit the *AF* was limited to 1.5.^156^ Hirata et al. developed pseudobilayer membranes that exhibit asymmetric water transport characteristics by selectively hydrolyzing one surface of a poly­(ethylene-*co*-vinyl acetate) (EVA) film.^157^ This treatment generated a gradient of vinyl alcohol and vinyl acetate residues along the transversal direction, yielding a hydrophilic, water-plasticizable layer on the hydrolyzed side, and a more hydrophobic EVA layer on the untreated side.^157^ The water permeability (*WP*) measured from the hydrolyzed surface exceeded that obtained in the opposite direction. This “flow reversal effect” was explained by the authors with the different extents of plasticization generated within the membrane in the two opposite transport directions.^157^ This hypothesis was corroborated by the finding that the *AF* increased with the thickness of the hydrolyzed layer, reaching a maximum value of 2.5.^157^

A particularly noteworthy system was reported in 1965 by Rogers, who modified poly­(ethylene) (PE) membranes by grafting these substrates in a graded manner with PVA. These membranes displayed asymmetry factors of up to 6.5 for water transport, among the highest reported to date (Table 1, Figure 15).^160^ In this study, one side of the PE films was exposed to vinyl acetate (VAc) vapors and subsequently subjected to high-intensity ionizing radiation to produce graded poly­(ethylene) membranes grafted with VAc.^104^ After the grafting reaction was complete, the vinyl acetate residues were hydrolyzed to vinyl alcohol, thus generating a compositional gradient of PVA grafts across the film thickness.^104^ At high *RH*, the water permeability measured in the PVA → PE direction largely exceeded the *WP* obtained in the opposite direction (Figure 15a).^104^ Interestingly, Rogers did not discuss the increase in the directionality observed at *RH* > 0.83, where the transport switched from symmetric (*AF* ∼ 1) to asymmetric (*AF* > 1) (Figure 15b). Instead, the study focused on the highly anisotropic permeation observed at elevated water vapor pressure, which was attributed to the higher water solubility in PVA than in PE.^104^ Although the PE-*g*-PVA membranes exhibited an asymmetry factor as high as 6.5, this value remains well below the theoretical prediction of *AF* approaching 10 determined by Petropoulos in his modeling studies.^69,102^ Moreover, the fabrication process of these compositionally graded membranes is remarkably complex and prohibitively time-consuming, requiring nearly 2 weeks for the hydrolysis step,^104^ which limits their scalability and practical applications. Finally, all the reactive processes discussed above for creating graded membranes share the limitation that the composition of the final membrane is difficult to control. This is likely one of the main reasons why most of these systems display very low *AF* values.

**Figure 15:**
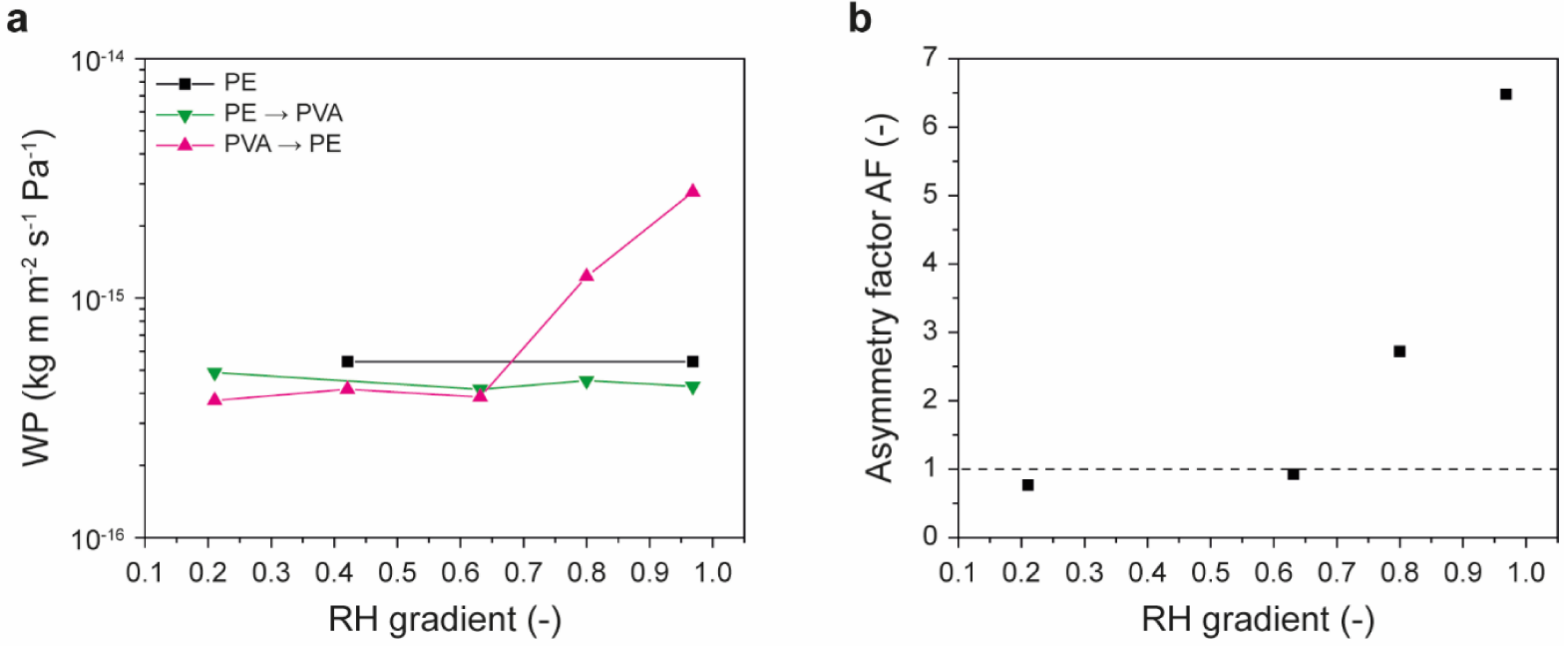
(a) Water permeability (*WP*) of poly­(ethylene) (PE) and compositionally graded PE-*g*-PVA membranes as a function of transport direction (i.e., PVA → PE and PE → PVA) and relative humidity (*RH*) gradient. (b) Asymmetry factor (*AF*) of the graded PE-*g*-PVA membranes as a function of *RH* gradient. The graphs show data from ref 104 that were replotted.

The lamination of two polymer films is arguably the simplest approach to producing spatially heterogeneous membranes. Notably, approximately half of the membranes reported in Table 1 have laminated structures. This group includes the membranes developed by Cassidy and colleagues, who studied the water transport properties of bilayer membranes fabricated by laminating commercially available elastomers.^159–161,164^ For all of the investigated systems, the asymmetry values remained below 1.8, suggesting that the *WPs* of the hydrophobic elastomers employed in these studies, i.e., ethylene-propylene-diene terpolymer (EPDM), chloroprene (CR), styrene–butadiene rubber (SBR), and nitrile butadiene rubber (NBR), vary only little upon exposure to moisture.^69,159–161^

As elaborated by Petropoulos, achieving significant directional permeation requires the use of components whose permeability shows a pronounced dependence on the permeant’s vapor pressure.^69^ Applied to the context of directional water transport, high *AF* values are observed for membranes containing components whose *WP* varies markedly with the water vapor pressure (Table 1), for example, the PA6 or PVA employed by Rogers (Figures 9,15).^32,104^ Creating PVA-containing membranes through lamination thus appears to be an attractive proposition. Such membranes were first reported by Matsuno and coworkers, who combined a PVA layer with hydrophobic layers of either poly­(vinyl acetate) (PVAc) (Figure 16) or poly­(ethylene terephthalate) (PET) (Figure 17) using lamination techniques.^103^ While PVAc and PET reference films each exhibit a relatively constant *WP*, the *WP* of PVA depends strongly on the applied RH gradient, so that the transport through the bilayers is directional. Interestingly, the relationship between water vapor pressure and the *WP* of PVA reported by Matsuno et al.^103^ appears to differ from the sigmoidal dependence observed by Hauser and McLaren^118^ and confirmed in more recent studies.^122,163^ This discrepancy, however, is only apparent and arises from the limited range of water vapor pressures investigated by Matsuno,^103^ which does not extend into the high-humidity regime where the water permeability of PVA saturates, but instead corresponds primarily to the intermediate region of the sigmoidal trend characterized by a pronounced increase in *WP*.^122,163^ A comparison shows that the neat PET films exhibit a much lower *WP* than the PVAc layers (Figure 16a, Figure 17a). PVAc is thus much less efficient in protecting the PVA layer from water uptake when the hydrophobic side of the bilayer membrane is exposed to moisture. Therefore, the maximum *AF* of the PVA/PET bilayer membranes (4) is considerably higher than that of the PVA/PVAc membranes (1.6) (Figure 16b, Figure 17b).^103^ Interestingly, Matsuno observed a maximum in the *AF* of the PVA/PET bilayers at *RH* ∼ 0.65, i.e., the crosspoint in *WP* between PVA and PET (Figure 17b).^103^ The subsequent decrease in directionality was attributed to the predominance of the PET layer: in the bilayers studied, the PET layer was thicker than the moisture-sensitive PVA layer, and its low *WP* governed the directionality, causing a maximum in the *AF* at this intermediate vapor pressure, even though the *WP* of PVA peaks at higher *RH*.^103^

**Figure 16:**
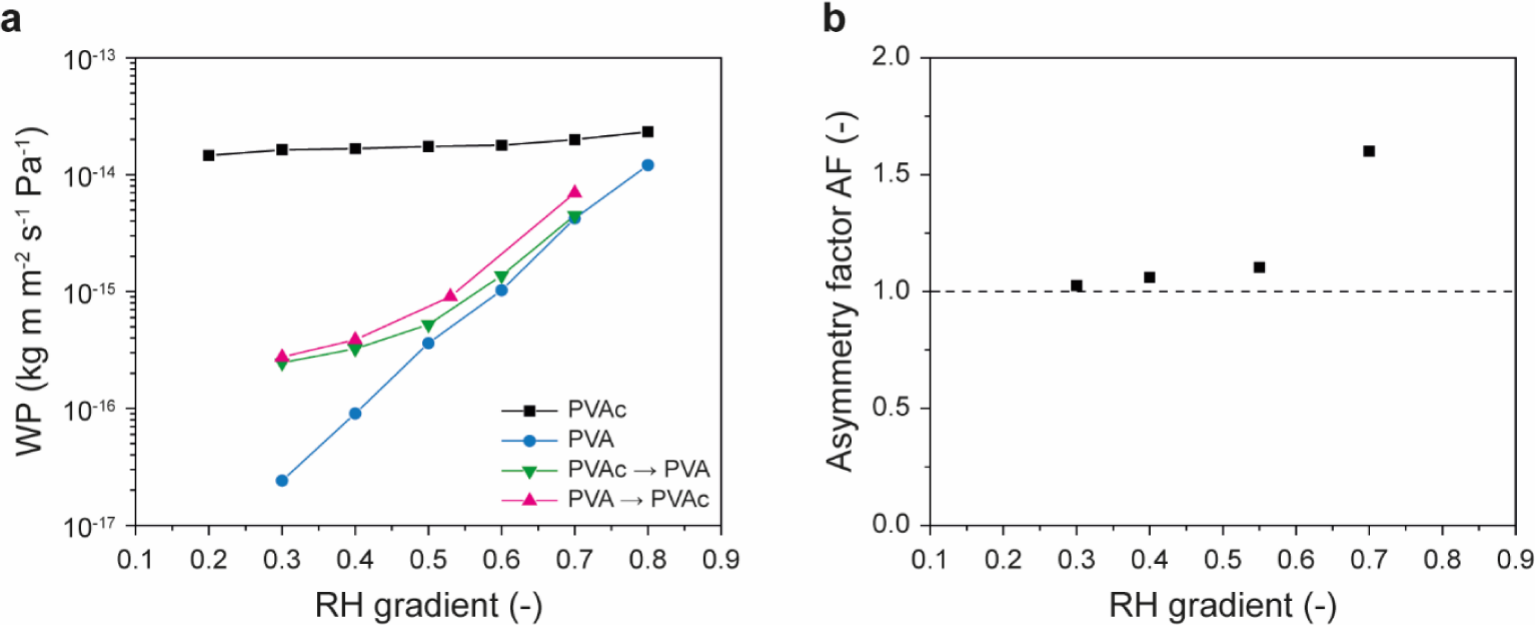
(a) Water permeability of poly­(vinyl alcohol) (PVA), poly­(vinyl acetate) (PVAc), and laminated PVA/PVAc membranes as a function of transport direction and relative humidity (*RH*) gradient. (b) Asymmetry factor (*AF*) of the PVA/PVAc laminated membranes as a function of *RH* gradient. The graphs show data from ref 103 that were replotted.

**Figure 17:**
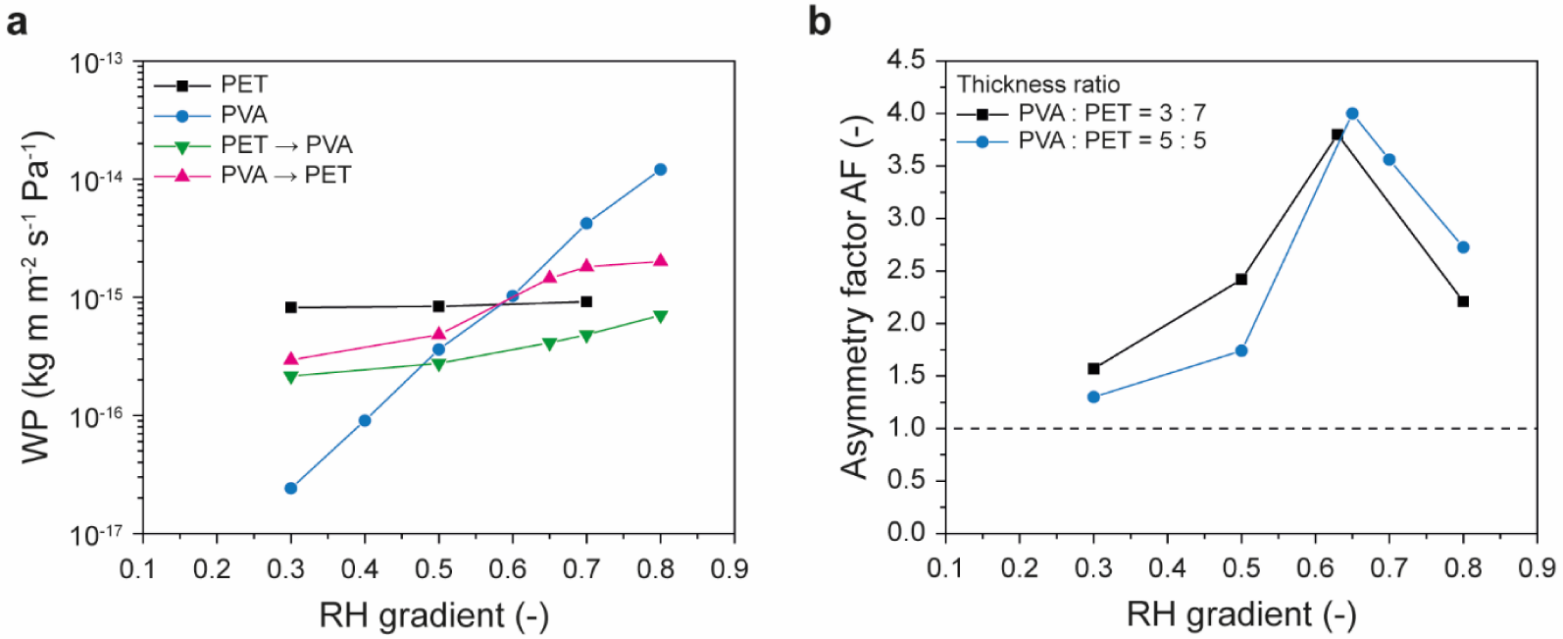
(a) Water permeability of poly­(vinyl alcohol) (PVA), poly­(ethylene terephthalate) (PET), and laminated PVA/PET membranes with thickness ratio PVA:PET = 5:5 as a function of relative humidity (*RH*) gradient and transport direction. (b) Asymmetry factor (*AF*) of the PVA/PET laminated membrane as a function of the applied *RH* gradient and the thickness ratio between the PVA/PET layers. The graphs show data from ref 103 that were replotted.

Using sorption and permeation data collected for PVA and PET reference films, Matsuno et al. developed a model for water transport across PVA/PET bilayer membranes, which closely reproduced the experimental *AF* values.^103^ This study represents a rare example of work that combines experimental characterization with theoretical modeling to elucidate the factors affecting directional water transport in laminated membranes. For example, the model predicts that the *AF* increases with the relative thickness of the PET layer, a trend that was confirmed experimentally by varying the PVA/PET thickness ratio (Figure 17b).^103^ Despite these insights into asymmetric permeation, the bilayer membranes were not subjected to mechanical characterization and displayed delamination under high water vapor pressure,^103^ leaving their structural integrity and mechanical robustness under application conditions in doubt.

PVA was recently used as the moisture-sensitive component to improve the performance of the above-discussed cuticle-inspired SBS/CNC membranes. An important limitation of the latter is the high crystallinity of the cellulose nanocrystals,^68,165^ which renders these nanoparticles virtually impermeable and confines water transport to the hydrophilic channels along the CNCs surfaces.^68,166^ To address this limitation, Grillo and Weder replaced the CNCs with PVA nanofibers.^158^ Compositionally graded membranes were prepared by electrospinning PVA nanofiber mats, which were then overcast with an SBS solution, affording SBS-PVA nanocomposite membranes with PVA- and SBS-rich sides. The resulting SBS-PVA nanocomposites exhibited not only asymmetric water permeation in the preferential direction from the PVA-rich to the SBS side of the membranes, but also displayed a switchable behavior in directionality, mimicking the feature observed in the plant leaf cuticles that inspired the nanocomposite structure (Figure 18).^68,158^ Under a low relative humidity gradient (*ΔRH* < 0.75), the water transport through the SBS-PVA nanocomposite membranes remains essentially symmetric (*AF* ∼ 1, Figure 18b), as under these conditions the PVA is not heavily plasticized (Figure 10).^122,163^ At higher *ΔRH*, swelling and plasticization of the PVA nanofibers induce a glassy-to-rubbery transition that triggers the switch to asymmetric transport (*AF* > 1, Figure 18b).^158^ It is interesting to note that in the SBS-PVA nanocomposites, the transition from symmetric to asymmetric transport occurs at a higher *ΔRH* than that observed by Matsuno and coworkers (Figure 16).^103^ This difference may be related to the fact that in the SBS-PVA nanocomposite membranes, the PVA nanofibers are embedded in the hydrophobic SBS matrix, so that a higher external *RH* is needed to plasticize the PVA.^158^

**Figure 18:**
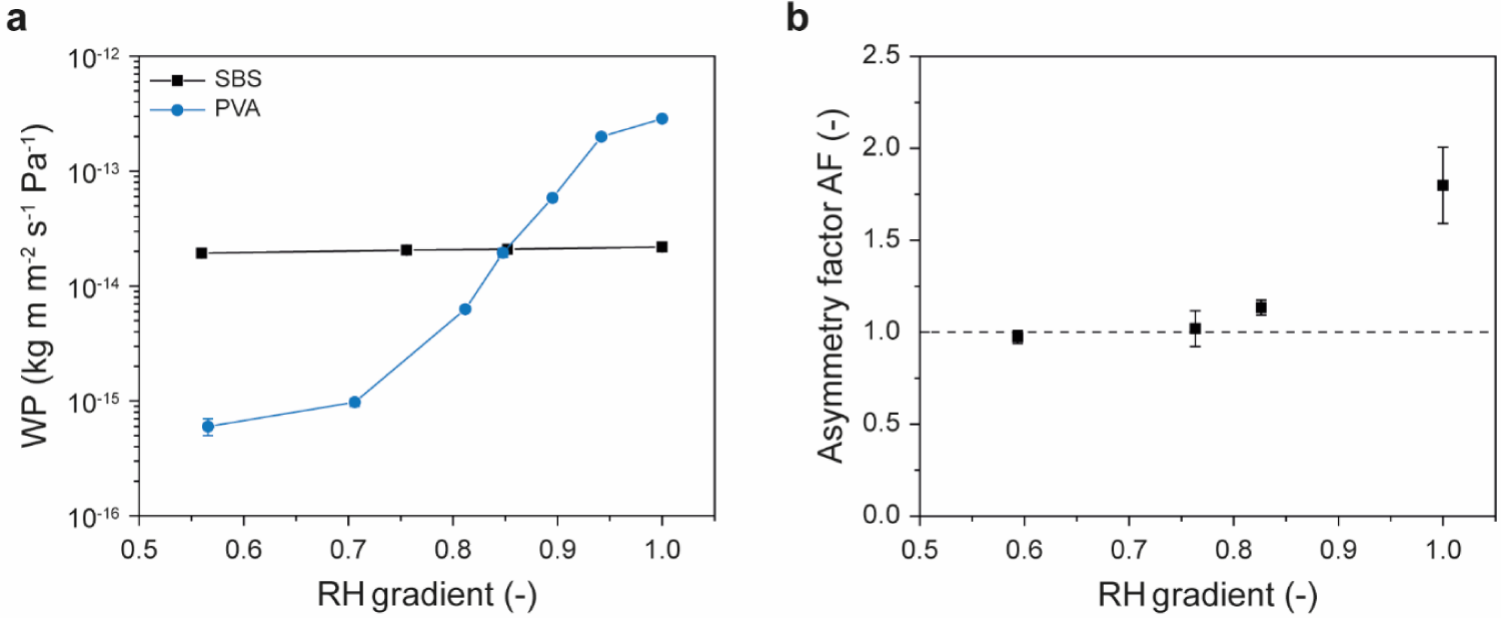
(a) Water permeability of neat poly­(vinyl alcohol) (PVA) and neat poly­(styrene)-*block*-poly­(butadiene)-*block*-poly­(styrene) (SBS) as a function of relative humidity (*RH*) gradient. (b) Asymmetry factor (*AF*) of SBS-PVA nanocomposite membranes as a function of *RH* gradient. The graphs show data from refs 122 and 158 that were replotted.

Using the resistance-in-series model, a theoretical framework widely applied to analyze mass transport through composite and laminated membranes based on the fundamental principles described above,^74,84,93,94^ the authors confirmed the *AF* to be limited by the architecture and predicted that a laminated structure comprising thick PVA and thin SBS layers would increase the asymmetry of water transport.^158^ In a follow-up study, Grillo and Weder thus investigated PVA-SBS bilayer membranes to confirm these calculations. Indeed, *AF* values of up to 5.8 were achieved in optimized geometries after systematic variation of the layer thicknesses,^122^ more than twice the values observed for compositionally graded membranes prepared from the same constituents.^158^ Although the theoretical advantage of laminated over graded structures in achieving asymmetric permeation had already been anticipated by Yamanaka in 1993,^110^ the direct experimental comparison provided by the two studies of Grillo and Weder^122,158^ provides compelling validation and offers further guidance of the rational design of membranes with highly directional water transport.

Beyond architecture alone, however, the treatment of the PVA-SBS bilayer membranes within the framework of the resistance-in-series model suggests that the main limitation of this platform is related to the relatively modest contrast in water permeability between SBS (*WP*_SBS_ = 2.1 × 10^–14^ kg m m^–2^ s^–1^ Pa^–1^) and fully hydrated PVA (*WP*_PVA_ = 2.9 × 10^–13^ kg m m^–2^ s^–1^ Pa^–1^).^122^ Indeed, the *WP* of SBS is more or less on par with that of PVAc (Figure 16a).^103^ To test whether a larger contrast could overcome this limit, Grillo and Weder recently reported dense laminated membranes combining PVA with glycol-modified poly­(ethylene terephthalate) (PETG), which replaced SBS as a more efficient water barrier material.^167^ Using a modeling approach based on Petropoulos’ theoretical framework,^69^ the authors used experimental data obtained from the characterization of the water transport properties of neat PVA and neat PETG reference films (Figure 19a) to predict the *AF*_M_ (where the subscript M indicates a modeled value of *AF*) of PVA/PETG bilayer membranes (Figure 19b).^163^ In their modeling, the authors focused specifically on an *RH* gradient of 1, as under these conditions, PVA and PETG exhibit the strongest contrast in *WP* (Figure 19a). The modeling identified a range of combinations for which the *AF* approaches 8 (Figure 19b).^163^ To test these predictions, the authors prepared several membranes, including bilayers consisting of a PVA layer with a thickness *l*_PVA_ = 200 μm and a PETG layer with a thickness *l*_PETG_ = 30 μm. Gratifyingly, the experimental *AF* value of 6.7 was only marginally lower than the modeled value of 7.9. The difference was attributed to the presence of a thin adhesive interlayer in the actual laminates, which was not included in the model but necessary to promote effective adhesion at the interface between the PVA and PETG layers. Thus, a 15 μm thin poly­(styrene)-*block*-poly­(ethylene-*ran*-butylene)-*block*-poly­(styrene)-*graft*-maleic anhydride (SEBS-MA) interlayer, containing reactive maleic anhydride groups, was compression-molded between the two membrane components, supposedly forming covalent bonds with functional groups on both PVA and PETG,^168–170^ thereby improving adhesion and enabling mechanically stable laminates.^163^ Despite this minor deviation, the experimental trends of the asymmetry factor closely matched the model predictions, and the *AF* of 6.7 equalizes the highest value reported to date (Table 1).

**Figure 19:**
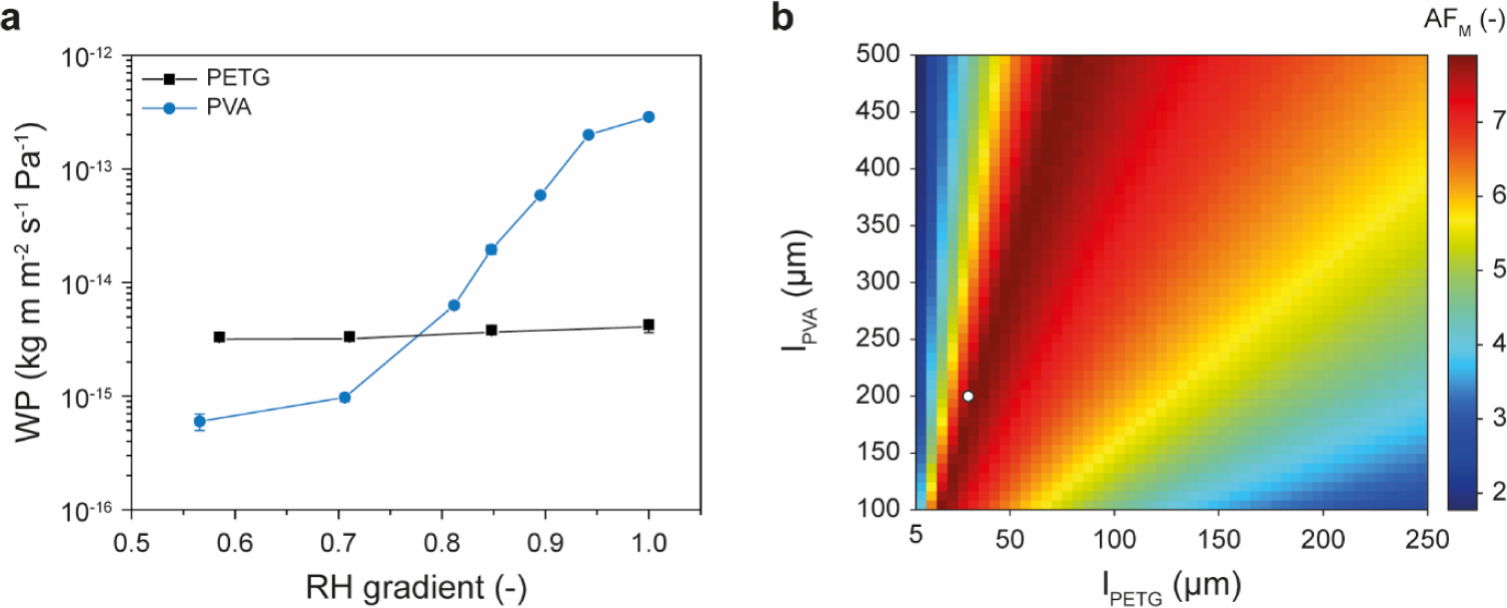
(a) Water permeability (*WP*) of poly­(vinyl alcohol) (PVA) and glycol-modified poly­(ethylene terephthalate) (PETG) films as a function of the *RH* gradient. (b) Modeled asymmetry factor (*AF*_M_) of PVA–PETG bilayer membranes at an *RH* gradient of 1, calculated as a function of the PVA (*l*_PVA_) and PETG (*l*_PETG_) layer thickness. The white circle denotes one optimal thickness combination yielding an *AF*_M_ of 7.9. Panel (a) shows data from ref 163 that were replotted. Panel (b) was reproduced with slight modifications from ref 163. Copyright 2026 American Chemical Society.

Directional moisture transport in dense multilayer membranes has been reported in recent work by Rattanaphong et al., who prepared laminated polymer blends via compression molding to generate a gradual change in composition in the transverse direction of pseudolaminated membranes.^162^ The blends were produced from the hydrophobic SBS and a three-component copolymer (terpolymer, TP) made from 2-hydroxyethyl methacrylate (HEMA), 2-hydroxyethyl acrylate (HEA), and 2-ethylhexyl methacrylate (EHMA), where the TP functions as the moisture-sensitive component.^162,171^ Originally developed for mechanically adaptive implants,^171^ the TP was mixed with SBS to form polymeric blends that exhibited humidity-dependent water permeability originating from the water-induced plasticization of the terpolymer.^162,171^ The authors used an SBS-TP blend with a moderate content of the hydrophilic component as an adhesion promoter between SBS and TP films, thus producing trilayer membranes that exhibited improved cohesion and an asymmetry factor of 4.0.^162^

These studies emphasize the importance of adhesion-promoting interlayers, whether introduced through polymer blending or chemical adhesion,^162,163^ in generating mechanically robust laminated membranes, which are otherwise prone to delamination.^172,173^ Collectively, these strategies offer versatile solutions for integrating hydrophilic and hydrophobic materials into mechanically robust laminates with tailored interfaces and highly asymmetric transport properties.

## 4 Potential Applications of Directional Water Transport in Dense Membranes

Following the overview of directional water transport observed in dense membranes, a key consideration is whether this feature retains technological relevance despite the inherently low permeation rates of nonporous systems.^67^ This drawback is frequently balanced by the higher selectivity of dense membranes and, in practical devices, by their incorporation into composite architectures with porous supports that enhance overall throughput. While directional water transport in porous membranes has been widely explored for separation and filtration applications,^174–179^ its potential for dense-membrane applications has received less attention and is therefore considered here.

In the context of pervaporation, a separation process in which a multicomponent liquid feed is fractionated by partial vaporization through a selective membrane,^180–182^ this limitation is commonly mitigated by coupling a thin dense selective layer with a porous substrate, which provides mechanical support while offering negligible resistance to mass transport.^180–183^ These so-called composite membranes are widely adopted in pervaporation due to their ability to achieve higher permeation fluxes and enhanced overall performance compared to standalone dense membranes.^180–183^ Enabling directional transport behavior in the dense selective layer of this configuration offers a further opportunity to improve separation efficiency, as proven by He and coworkers.^184^ In their study, the authors coated a porous poly­(vinylidene fluoride) (PVDF) substrate with a mixed-matrix membrane (MMM) composed of dense poly­(dimethylsiloxane) (PDMS) and carbon nanotubes (K-CTN), the latter being surface-functionalized with a silane coupling agent to enhance compatibility with PDMS.^184^ By tailoring the fabrication method, He et al. were able to control the spatial distribution of K-CTN within the dense PDMS layer, achieving either homogeneous dispersion or selective enrichment at the top or bottom side.^184^ When evaluated for bioethanol recovery via pervaporation, the composite membranes with asymmetric filler distribution displayed directional transport behavior, and the total water/ethanol flux was significantly higher when K-CTN were concentrated at the surface as opposed to the bottom of the PDMS layer.^184^ Among all composite membranes, comprising a porous PVDF substrate with either pristine PDMS or PDMS containing K-CTN dispersed homogeneously or asymmetrically, the configuration featuring K-CTN at the PDMS top surface had better pervaporation performance, illustrating how asymmetric filler distribution in the dense layer can induce directional transport of water and ethanol and enhance overall mass transfer.^184^

Another promising application of directional water transport in dense membranes is their integration with fabrics to enhance the performance of waterproof breathable materials (WBMs).^185^ In the context of WBMs, two primary configurations of membranes are adopted: monolithic (i.e., nonporous) hydrophilic membranes and microporous membranes laminated onto a protective, hydrophobic fabric.^185–187^ Water transport across monolithic membranes occurs through a solution–diffusion mechanism, whereas in microporous membranes it is governed by capillary transfer within the pore network.^185,188^ Introducing asymmetric water transport behavior into monolithic membranes could improve breathability by enabling preferential vapor transmission from the skin to the outer environment while maintaining impermeability to liquid water.

In addition to its relevance in breathable, functional clothing, directional water transport holds significant potential for biomedical applications, particularly in the design of advanced wound dressings. In this context, moisture management is crucial for creating a protective and yet balanced environment that supports optimal healing. An ideal wound dressing should therefore achieve a delicate balance between two opposing requirements: maintaining sufficient hydration to promote tissue regeneration while preventing excessive fluid accumulation.^189–191^ Typically, porous materials are employed in wound dressing to facilitate moisture regulation and partially mimic the skin’s structure.^190–192^ However, their interconnected pore networks can disrupt hydration balance and compromise barrier integrity by facilitating bacterial infiltration. In contrast, dense occlusive dressings effectively prevent contamination, but may induce exudate retention and hinder healing.^191^ To overcome these limitations, laminated membranes combining a dense outer layer and a porous inner layer have emerged as promising wound dressing designs.^193^ In these bilayer structures, the dense layer acts as a barrier against bacterial invasion and provides mechanical strength, while the porous layer facilitates exudate drainage and supports cell proliferation, collectively creating a controlled healing environment.^192,193^ Integrating directional water transport into the dense layer could improve moisture management by promoting controlled vapor removal, while preserving its barrier function against bacteria and fluid penetration.

Another noteworthy biomedical application of directional water transport, alongside its role in wound dressing design, lies in advanced drug delivery systems. In this context, implementing directional water transport mechanisms within tablet film coatings could offer a sophisticated and effective strategy for achieving controlled release of active pharmaceutical ingredients, thereby enhancing therapeutic precision.^194–196^

Extending this concept beyond biomedical applications, coatings engineered with directional water-transport properties could also be relevant for the preservation of historic monuments, whose materials are highly susceptible to moisture-induced degradation.^197,198^ Conventional protective coatings are typically homogeneous and, while they act as barriers against external moisture, they often trap preexisting water within the material, thereby accelerating deterioration.^198^ In contrast, coatings exhibiting asymmetric water transport behavior offer a promising strategy for regulating moisture exchange, allowing outward diffusion while limiting inward penetration, and provide a tunable and more effective protective function.^198^ Notably, Li et al. recently explored the application of directional water transport to conserve silicate relics, demonstrating its effectiveness in mitigating damage caused by salt efflorescence.^199^

Another promising domain for the application of dense membranes with asymmetric water transport is smart and functional food packaging. Functional packaging materials are generally classified as either sealed or breathable, depending on their structural characteristics and moisture exchange capabilities.^200^ While sealed packaging effectively protects products from external contaminants (e.g., oxygen, moisture, and microorganisms), it is often unsuitable for perishable goods, such as fruits and vegetables, which continuously release metabolic byproducts.^200^ Breathable packaging solutions can overcome these limitations by controlled removal of metabolic gases and water vapor while preserving the product’s quality and freshness.^200^ Thus, the preservation of freshness and safety of food products strongly depends on the microenvironment within the package, with humidity control emerging as a key factor in extending shelf life and preventing deterioration.^201^ In this context, directional water transport properties offer a promising strategy for developing fresh-keeping packaging materials that can regulate the humidity inside the package, thereby mitigating food spoilage.^201^ A noteworthy study in this area was reported by Hirata and coworkers, who fabricated pseudobilayer membranes through the partial hydrolysis of poly­(ethylene-*co*-vinyl acetate) (EVA) on one surface, resulting in the localized formation of poly­(ethylene-*co*-vinyl alcohol).^157^ As previously discussed, these membranes exhibited directional water transport, primarily attributed to the different extent of plasticization on the hydrolyzed side depending on the transport direction.^157^ In addition to this anisotropic behavior, the membranes displayed enhanced H_2_O/O_2_ and H_2_O/CO_2_ selectivity, particularly in the direction from the hydrolyzed to the nonhydrolyzed surface.^157^ Considering that effective preservation of fruits and vegetables requires packaging films with high water vapor selectivity to regulate respiration and minimize anaerobic fermentation, the system developed by Hirata et al. demonstrated strong potential for smart food packaging applications.^157^ To fully realize the potential of directional water transport, materials exhibiting this property must be designed and assembled in an optimized configuration to ensure their practical applicability across a range of applications, from innovative packaging to separation processes.^157,184^ In the context of packaging, Del Nobile et al. theoretically demonstrated that bilayer membranes composed of cellophane and polyethylene (PE) exhibit different overall water permeability depending on the side that is exposed to high humidity.^113^ This behavior was attributed to the moisture-dependent *WP* of cellophane, which increases with relative humidity.^113^ In another theoretical study, Jakobsen and Risbo showed that incorporating moisture-sensitive materials within multilayer packaging structures can also affect the oxygen permeability under varying humidity conditions.^117^ In their investigation of laminates consisting of ethylene-vinyl alcohol copolymer (EVOH) and PE, they further showed that asymmetric configurations can more effectively preserve modified atmospheres in packaged food.^117^ Collectively, these findings emphasize that the barrier properties of multilayer membranes can be precisely and directionally controlled through careful selection of materials and their structural arrangement.^113,114,117^ This strategy is also applicable in other membrane technologies, such as pervaporation, where the lamination of different materials can optimize separation performance.^182,184,202^

## 5 Summary and Outlook

The directional transport of water is a remarkable example of evolutionary optimization, enabling organisms to efficiently collect, retain, and regulate water exchange with their surroundings.^1–6^ This phenomenon is particularly evident in plant leaf cuticles, which are dense, compositionally graded membranes that can adapt their water permeability to ambient humidity.^26,38,39,68^ These biological membranes exhibit switchable asymmetric water transport, characterized by preferential inward water flux under dry external conditions and a transition to symmetric water permeability under humid environments.^63,68^ This humidity-responsive behavior arises from the interplay between the water-induced plasticization of cutin and the compositionally graded architecture, which together modulate both the magnitude and directionality of water transport.^68^

Membrane theory identifies three essential conditions for this effect: (1) spatial heterogeneity across the membrane thickness, (2) at least one element with a permeability that depends on the vapor pressure of the permeant, and (3) an external vapor-pressure gradient that induces the plasticization of the permeant-sensitive component.^32,67^ When these criteria are met, asymmetric permeation emerges. Despite extensive theoretical and modeling efforts aimed at understanding and optimizing directional transport,^69,102,107–112^ experimental validation remains limited.^67,103^ Bridging this gap between theory and experiment is essential for translating directional water transport into functional membrane technologies.

Recent progress in asymmetric polymer membranes highlights the importance of both material selection and membrane architecture.^122,158,163^ In particular, combining hydrophilic, humidity-responsive polymers with hydrophobic, water-barrier materials has proven highly effective in generating pronounced directional transport. Laminated architectures generally outperform compositionally graded structures,^122^ as they allow more precise control over composition and transport directionality. At the same time, interfacial compatibility between dissimilar layers is a critical factor, as poor adhesion can compromise both mechanical integrity and transport performance. Strategies such as reactive interlayers or polymer blending offer promising routes to enhance interfacial stability while preserving asymmetric transport functionality.^162,163^

Looking forward, the technological relevance of directional water transport must be critically assessed in application-oriented contexts. Dense asymmetric membranes are particularly attractive for moisture management^185,200,201^ and separation processes,^153,184^ such as pervaporation, where selective and controllable water transport is central to performance. As several moisture-sensitive polymers are already commercially employed in membrane technologies,^180–182^ the integration of directional transport concepts offers a realistic pathway toward advanced membrane systems. Exploiting direction-dependent and humidity-responsive permeability may enable new functionalities, including adaptive process control and improved separation efficiency.^153,184^

Further advances will benefit from the close integration of experimental studies with predictive modeling. Models that link single-layer permeability data to multilayer membrane performance offer powerful tools for rational design, enabling informed selection of material combinations and architectures to maximize asymmetry. Ultimately, bioinspiration, combined with progress in polymer chemistry, interfacial engineering, and transport theory, is expected to drive the development of mechanically robust, adaptive asymmetric membranes with tunable directional water transport for next-generation technologies.
